# Public preference for COVID‐19 vaccines in China: A discrete choice experiment

**DOI:** 10.1111/hex.13140

**Published:** 2020-10-06

**Authors:** Dong Dong, Richard Huan Xu, Eliza Lai‐yi Wong, Chi‐Tim Hung, Da Feng, Zhanchun Feng, Eng‐kiong Yeoh, Samuel Yeung‐shan Wong

**Affiliations:** ^1^ The Jockey Club School of Public Health & Primary Care Faculty of Medicine The Chinese University of Hong Kong Hong Kong SAR China; ^2^ Centre for Health Systems and Policy Research The Chinese University of Hong Kong Hong Kong SAR China; ^3^ School of Pharmacy Tongji Medical College Huazhong University of Science & Technology Wuhan China; ^4^ School of Medicine and Health Management Tongji Medical College Huazhong University of Science & Technology Wuhan China

**Keywords:** Chinese public, COVID‐19 pandemic, discrete choice experiment, vaccine, willingness to pay

## Abstract

**Background:**

As the coronavirus disease 2019 (COVID‐19) pandemic is sweeping across the globe, there is an urgent need to develop effective vaccines as the most powerful strategy to end the pandemic. This study aimed to examine how factors related to vaccine characteristics, their social normative influence and convenience of vaccination can affect the public's preference for the uptake of the COVID‐19 vaccine in China.

**Methods:**

An online discrete choice experiment (DCE) survey was administered to a sample of China's general population. Participants were asked to make a series of hypothetical choices and estimate their preference for different attributes of the vaccine. A mixed logit regression model was used to analyse the DCE data. Willingness to pay for each attribute was also calculated.

**Results:**

Data of 1236 participants who provided valid responses were included in the analysis. There was strong public preference for high effectiveness of the vaccine, followed by long protective duration, very few adverse events and being manufactured overseas. Price was the least important attribute affecting the public preference in selecting the COVID‐19 vaccine.

**Conclusions:**

The strong public preferences detected in this study should be considered when developing COVID‐19 vaccination programme in China. The results provide useful information for policymakers to identify the individual and social values for a good vaccination strategy.

**Patient or Public Contribution:**

The design of the experimental choices was fully based on interviews and focus group discussions participated by 26 Chinese people with diverse socio‐economic backgrounds. Without their participation, the study would not be possible.

## 1 INTRODUCTION

As of 24 August 2020, the novel severe acute respiratory syndrome coronavirus 2 (SARS‐CoV‐2) virus has infected more than 23 million people in 216 countries and regions, with a case fatality ratio (CFR) of approximately 3.4%.^1^ Currently, there is no effective treatment for this disease, and relaxation of effective non‐pharmaceutical interventions often leads to resurgence of community outbreaks.^2^, ^3^, ^4^, ^5^ Thus, a vaccine seems to be the only solution to this problem. As vaccines are regarded as the most cost‐effective way of controlling infectious diseases, there are attempts to develop a coronavirus disease 2019 (COVID‐19) vaccine rapidly to catch up with the rate of the pandemic's spread.^6^ On 20 July 2020, the so‐called Oxford vaccine (ChAdOx1 nCoV‐19) was announced as a front runner among 24 candidate vaccines in clinical evaluations worldwide. The reason is that it was proven in a stage 1/2, single‐blind, five‐site, randomized clinical trial that the vaccine could spike up antibodies and create a safe immune response in the body.^7^

However, the path to introducing a new vaccine to the market can be politically and economically complicated. The COVID‐19 vaccine is no exception. Although insights and opinions of different stakeholders—such as policymakers and medical professionals—might affect the vaccine's uptake to some extent,^8^ the most essential factor for any vaccination programme's successful adoption is the public's acceptance. Factors such as individual characteristics (eg high‐risk occupations and socio‐economic vulnerability) and disease‐specific characteristics (eg morbidity) play an important role in the individual's decision‐making process to select vaccination programmes.^9^, ^10^ A reasonable strategy should consider both the provider's affordability and consumer's preference. However, currently, studies investigating these factors and their effect on the public's preference in selecting the COVID‐19 vaccine are limited and fragmented. Obtaining such information is important for policymakers to understand the individual and social values to optimize strategies and design potential vaccination campaigns to address COVID‐19 as well as for pharmaceutical companies to estimate the expected benefit when managing the vaccine's development.^11^ Moreover, the acceptance rate for a possible vaccine also reflects the public's willingness to be vaccinated. Thus, this study aimed to examine how the relative importance of factors related to vaccine characteristics, the social normative influence and convenience of vaccination affects the public's preference for the uptake of the COVID‐19 vaccine in China.

China was selected as the research location for two reasons. First, China is one of the leading candidates in the global vaccine development contest, as three of its vaccines were reported to have already completed the phase 2 clinical trials. At the end of June 2020, China's state‐run CanSino Biologics announced that their vaccine candidate demonstrated a 'good safety profile' with high levels of immune response in patients, and it is highly probable to be authorized for urgent use, including for front‐line medical professionals, at the end of this year.^12^ Thus, a broader commercial use of the vaccine may not be too far off. Second, China's Wuhan City is regarded as the epicentre of the COVID‐19 pandemic. Moreover, China is one of the few countries recovering from the pandemic via careful manoeuvering to return to normal. Nevertheless, the pandemic's impact at the physical, psychological, social and economic levels is extensive and long‐lasting. Hence, this study provides empirical evidence to identify the optimal COVID‐19 vaccination programme for promoting the vaccine's uptake among the general Chinese population and indicates key attributes for consideration when other countries start to develop their own COVID‐19 vaccination programmes.

## 2 METHODS

**FIGURE 1:**
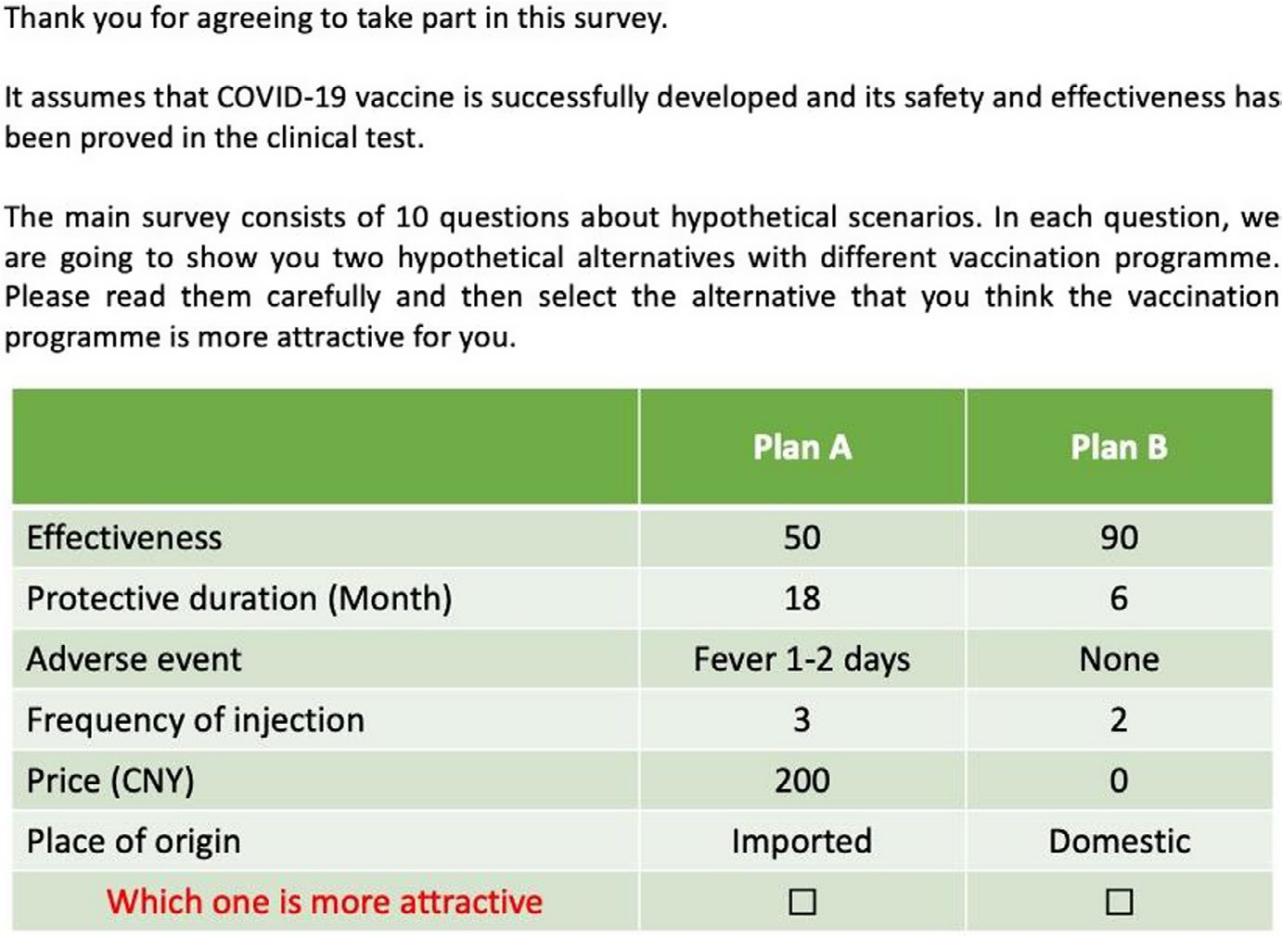
An example of choice set

To explore public preferences for the COVID‐19 vaccination programme, we used a discrete choice experiment (DCE) task administered online.^13^ For each choice task, there were two options of hypothetical vaccination programme alternatives. To ensure all respondents make a choice and to detect their preference, no opt‐out alternative was provided. An example choice set is provided in Figure 1. The major benefit of using the stated preference method is that it allows us to understand and capture the public's preference for vaccination programmes that do not currently exist but could in future be available.

### 2.1 Selection of attributes and levels

The criteria defined by Norman et al^14^ were followed to develop the attributes and levels of our DCE questionnaire. According to these criteria, first, all levels and their combinations should be reasonable. Second, all levels and their combinations should be familiar to respondents in their current practice. Third, heterogeneity of the levels should be fully considered in the design to ensure the respondents can make some trade‐offs between them.

The attributes and levels were selected using a standard iterative process adopted by previous studies that used DCE.^13^, ^15^, ^16^ First, the research team conducted a comprehensive literature review with articles extracted from the Cochrane Library, Web of Science, MEDLINE and EMBASE (1950‐2019), including academic articles using a variety of research methods such as quantitative and/or qualitative study designs, systematic reviews and randomized clinical trials, and the other government reports and policy briefings from Google, to explore important factors that affect the public's willingness and attitude when making decisions on vaccination uptake. The search target was not limited to the COVID‐19 and other pneumonia vaccines, but extended to other fields such as the influenza vaccine. Two researchers independently completed the literature review. All the team members discussed the findings, and four attributes—effectiveness,^17^, ^18^, ^19^, ^20^ protective duration,^17^, ^19^, ^20^, ^21^ adverse events^22^, ^23^, ^24^ and frequency of injections^25^, ^26^—were confirmed that potentially important for developing our DCE questionnaire. Then, on the basis of the findings from the literature review, six one‐to‐one interviews (three males and three females, aged between 28 and 62 years) and three focus group interviews with six to eight participants in each group (20 participants in total) as a sample of the Chinese general population were conducted to investigate their views and perspectives about the attributes of an 'ideal' COVID‐19 vaccine and its effectiveness at different levels. Several new insights were derived from the qualitative interviews. A new attribute—place of origin—that was considered to indirectly reflect the quality of the vaccine was added based on the interview results. The expression and explanations of the attributes and levels were modified according to the interviewees’ suggestions and comments. Third, a team of experts from relevant areas (clinical medicine, methodology, infectious disease and policy, five persons in total) was invited to discuss the findings from the previous steps. Two rounds of discussion were organized, experts and research team worked together to discuss how to modify and refine the attributes and levels to meet our criteria. Considering the majority of the adult vaccines are not free of charge in China, a cost parameter, which reflected the price of COVID‐19 vaccination programme, was included in the DCE questionnaire. An optimal mode of presenting choice sets to the respondents was also determined by experts to ensure the maximization of the face validity—the extent of a measure to capture what it is intended to measure—of the choice task.^27^ Thus, finally, based on the literature review, general public interviews and expert discussions, six attributes with two to six levels for each were developed. The final set of attributes and levels is presented in Table 1.

**TABLE 1:** The attributes and levels of the discrete choice experiment study

Attribute	Levels
Effectiveness (%)	50
	70
	90
Duration of protection (month)	6
	12
	18
Adverse event	No reactions
	Local reactions such as redness and/or swelling at the site of vaccination for 1‐2 d
	Fever 1‐2 d
The total number of injections	1
	2
	3
Price (CNY)	0
	200
	400
	600
	800
	1000
Origin of product	Imported product
	Domestic product

Abbreviation: CNY, Chinese Yuan.

### 2.2 Experiment and questionnaire design

A full‐factorial design using all the attributes and levels results in 3 × 3×3 × 3 × 6 × 2 = 972 possible profiles, which provide 471 906 pairwise choice sets for selection. Using the STATA software (StataCorp LLC), 40 pairwise choice sets were constructed using a D‐optimality algorithm with the attribute coefficient set to zero. Only the main effect was estimated in this study. There is no standard guidance in the literature on the optimal number of DCE tasks that each respondent should complete. In this study, 40 choice sets were randomly assigned to four blocks, each of which had 10 choices. All of the choice sets were checked for plausibility, and no manual alteration of the design was required.

To assess the internal validity of DCE questions, a choice set with dominated pairs was presented (trap question) to all respondents.^28^ In that choice task, one alternative was unambiguously better than the other alternative for all attributes. Only DCE data that the respondents correctly selected as the dominated alternative from this choice set would be included in the analysis.

The survey questionnaire's first page provided the study information. Participants were not allowed to continue the survey until they had read details of the informed consent and agreed to participate in the study by clicking the 'Agree' button at the bottom of the page. The questionnaire consisted of three sections. In the first section, respondents were advised that they would be providing information about their health conditions; their knowledge of, attitudes towards and experience with the COVID‐19 pandemic; and their previous experience of vaccination. In the second section, a dominated DCE task was presented to the respondents to check whether they understood the DCE design and provided a plausible answer. To reduce systematic selection biases, one of the four blocks with 10 standard DCE tasks for each was randomly chosen by the survey software and presented to the respondents. Each respondent was confronted with one block of 10 DCE questions. After completing the DCE questions, respondents were asked to provide a subjective assessment of the difficulty of the questions on a five‐point Likert scale ranging from 'very hard' to 'very easy'. The last part of the questionnaire included questions to collect information on the respondents’ demographics, socio‐economic status (SES) and an overall evaluation of their mental health status using the Patient Health Questionnaire‐2.^29^

Before the formal study, a pilot DCE survey was conducted. A convenience sample of 10 members of the general public was invited to participate in the online survey. First, they were asked to complete the questionnaire independently through the same online platform as in the formal survey. Second, an interview was conducted immediately by the first two authors to understand their comments and suggestions on the survey and the approach.

### 2.3 Sample and survey administration

The survey was managed online via Wenjuanxing (WJX, https://www.wjx.cn/), the biggest online survey company in China, between June and July 2020. The questionnaire was developed by the research team using WJX’s survey design software built on its online survey platform. Participants were recruited by the same survey company via its members on the online panel. An online panel is a form of access panel and includes 'a sample database' consisting of registered participants who agree to occasionally participate in Internet‐based studies; these have become increasingly prevalent in academic research.^30^, ^31^ In this study, the inclusion criteria of participants was ≥18 years; Chinese citizen; and stay at China during the last six months. Although previous studies have indicated that using the Internet to collect data might lead to certain forms of interviewer bias, a growing number of researchers agree that web‐based surveys, which provide a quick and cost‐effective way to collect DCE data, are often preferred by participants than surveys administered by interviewers.^13^, ^32^ Thus, this study adopted a web‐based survey considering that nearly 0.8 billion Chinese people currently have access to the Internet. The Survey and Behavioural Research Ethics Committee of the Chinese University of Hong Kong approved the study protocol and informed consent (Reference No.: SBRE‐19‐690).

### 2.4 Data analysis

Descriptive statistics were used to present the participants’ demographics, SES, and physical and mental health status. The random utility theory provides the theoretical foundation for analysing the DCE data. The public's utility (U) associated with a particular vaccination programme had two components: the deterministic component (V) and the stochastic component (ε).

The model of utility for an individual *n* associated with vaccination programme *i* can be estimated as

$$\begin{array}{cc} U_{n} & =V_{n}+\varepsilon_{n}\\ & =\beta_{1}*{\text{Effectiveness}}_{70}+\beta_{2}*{\text{Effectiveness}}_{90}+\\ & \;\beta_{3}*{\text{Duration}}_{12}+\beta_{4}*{\text{Duration}}_{18}+\\ & \;\beta_{5}*{\text{Adverse}}_{\text{moderate}}+\beta_{6}*{\text{Adverse}}_{\text{no}}+\\ & \;\beta_{7}*{\text{Injection}}_{2}+\beta_{8}*{\text{Injection}}_{1}+\\ & \;\beta_{9}*{\text{Production}}_{\text{import}}+\beta_{10}*\text{Price}+\varepsilon_{n}. \end{array}$$

The DCE data were binary, where '1' indicates that the alternative plan was chosen and '0' means that the other alternative plan was chosen. All attributes were dummy‐coded, and the coefficients of each level were estimated in the model and summarized to reflect the overall utility for each profile. The mixed logit regression (MXL) model was used to analyse the DCE data, as it estimates a distribution around each mean preference parameter to avoid potential bias of the estimated mean preference weights caused by unobserved heterogeneity.^33^ The attribute of 'price' was specified as a continuous variable to facilitate the calculation of willingness to pay (WTP), which is the monetary value that people place on different attributes of the vaccination programme.

We calculated the utility value and relative predicted probabilities for all profiles of the experimental design, which allowed us to compare profiles that are more likely to be chosen by respondents with profiles that are less likely to be chosen.^34^ This allowed us to convey the DCE results as easily understood information for the general public and policymakers. Subgroup analysis was also conducted to estimate the public's preference heterogeneity regarding vaccination programmes in terms of the respondents’ gender (men/women), family registry (urban/rural), parenting (yes/no) and personal vaccinated experience (yes/no). All statistical analyses were conducted using R (R Foundation, Austria) and STATA. The *P*‐value was set at ≤.05.

## 3 RESULTS

### 3.1 Responders’ characteristics

**FIGURE 2:**
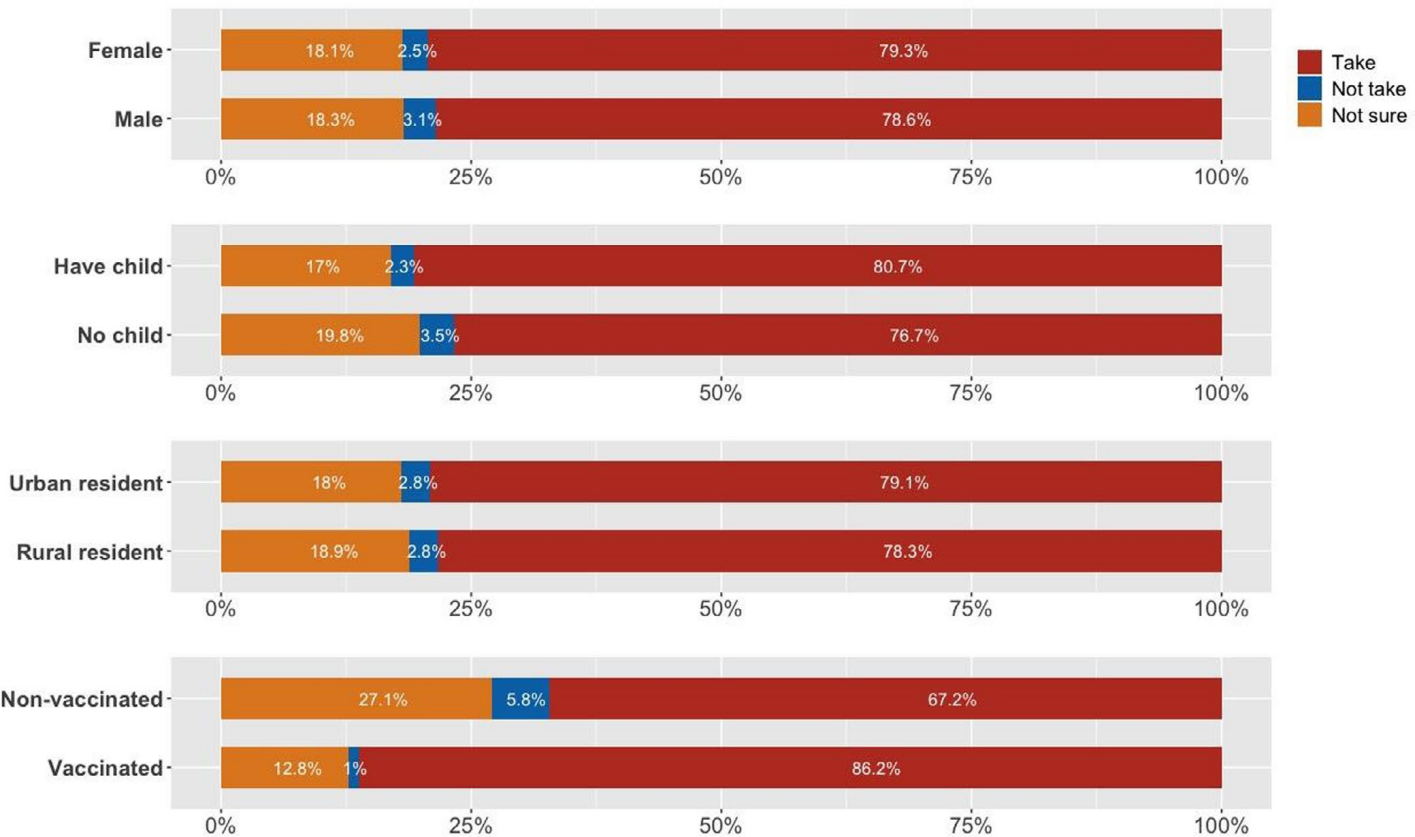
Respondents’ attitude towards COVID‐19 vaccine uptake

A total of 1694 individuals participated in the online survey, among whom, 177 did not consent or complete the questionnaire, 240 did not answer the trap item correctly, and 41 indicated the DCE questions are hard or very hard to be understood. These 458 answers were excluded from the analyses. Thus, data from 1236 individuals (72.96%) were elicited for our analyses. Four versions of the DCE questionnaire were completed by an approximately equal numbers of respondents (Appendix Table A1). Nearly half of the respondents were men, and the mean age was 30.27 years. The majority was married (60.6%) and lived in an urban area (72.18%). Most respondents were employed full time (78.4%), lived with their families (85.84%) and were protected by some form of medical insurance (98.62%). More than 80% reported a personal monthly income greater than the median monthly income in China (around 2200 Chinese Yuan [CNY]; 1 CNY = 0.14 USD) (Table 2). Compared with the national census data, our sample showed a similar sex ratio and proportion of medical insurance coverage, but higher educational attainment and proportion of living in the urban area. Figure 2 demonstrates that the respondents who were women (79.3%), had children (80.7%), lived in an urban area (79.1%) and were vaccinated in the past (86.2%) showed a more positive attitude towards taking the COVID‐19 vaccine.

**TABLE 2:** Characteristics of all respondents (n = 1236)

	Sample		General public^a^
	n	%	%
Sex			
Male	607	49.11	51.1
Female	629	50.89	48.9
Age, mean (SD)	30.27	7.66	
Educational level (aged > 18)			
Secondary and below	176	14.24	85.9
Tertiary and above	1060	85.76	14.1
Marital status			
Unmarried	480	38.83	18.2
Married	749	60.60	74.1
Divorced/widow	7	0.57	7.7
Family register			
Urban area	954	77.18	59.9
Rural area	282	22.82	41.1
Number of children			
0	556	43.54	
1	600	46.99	
≥2	121	9.47	
Living status			
Live along	104	8.41	
Live with family	1061	85.84	
Live with friends	65	5.26	
Others	6	0.48	
Working status			
Full‐time employed	969	78.4	96.3
Part‐time employed	44	3.56	
Farming	11	0.89	
Students	194	15.7	
Housewife	2	0.16	
Retired	4	0.32	
Unemployed	12	0.97	
Medical insurance			
Yes	1219	98.62	96.5
No	17	1.38	3.5
Personal income (CNY/month)			
<1000	98	7.93	
1000‐1999	85	6.88	
2000‐2999	71	5.74	
3000‐3999	112	9.06	
4000‐4999	85	6.88	
5000‐5999	130	10.52	
6000‐6999	123	9.95	
7000‐7999	102	8.25	
8000‐8999	138	11.17	
9000‐9999	87	7.04	
≥10 000	205	16.59	

Abbreviations: CNY, China Yuan; SD, standard deviation.

^a^ Based on China Statistical Yearbook 2018.

### 3.2 Results of the main effect model

Table 3 shows that the order and signs of all the attributes were as expected, and the coefficient of the attributes, except for the 'number of injections = 2', was statistically significant. The results demonstrated that the most important attribute was effectiveness. The coefficient of '90% effectiveness' was 3.138 (*P* < .001), followed by that of '70% effectiveness' (b = 1.416, *P* < .001). Although the COVID‐19 vaccine's price had a negative and significant effect on the respondents, it did not appear to be as important as the other attributes (b = −0.002, *P* < .001). Respondents’ preference for choosing a COVID‐19 vaccination programme increased with a longer protected duration but decreased with more adverse events and higher frequency of injections. In addition, we found that the place of manufacturing of the COVID‐19 vaccine affected the respondents’ preference—imported vaccine generated a higher utility score (b = 0.178, *P* < .001).

**TABLE 3:** Main effects model and WTP (n = 1236)

	Coefficient (SE)	*P*‐value	SD (SE)	*P*‐value	WTP	95% CI	
Effect 70%	1.416 (0.047)	<.001	−0.201 (0.16)	.211	878.879	790.626	967.131
Effect 90%	3.138 (0.093)	<.001	1.739 (0.091)	<.001	1948.158	1766.113	2130.204
Duration 12 mo	0.491 (0.041)	<.001	0.053 (0.074)	.473	305.018	252.072	357.964
Duration 18 mo	0.719 (0.05)	<.001	0.409 (0.092)	<.001	446.663	379.633	513.693
Moderate adverse event	0.471 (0.044)	<.001	0.286 (0.1)	.004	292.175	236.172	348.178
No adverse event	1.042 (0.056)	<.001	0.93 (0.065)	<.001	647.029	565.525	728.533
Injection 2 times	0.059 (0.044)	.177	−0.019 (0.109)	.859	36.791	−16.956	90.537
Injection 1 time	0.159 (0.042)	<.001	0.317 (0.083)	<.001	98.417	47.56	149.273
Imported	0.178 (0.03)	<.001	−0.081 (0.117)	.492	110.46	72.635	148.284
Price	−0.002 (0)	<.001	0.002 (0.001)	<.001			

Abbreviations: 95% CI, 95% confidence interval; SE, standard error; SD, standard deviation; WTP, willingness to pay.

**FIGURE 3:**
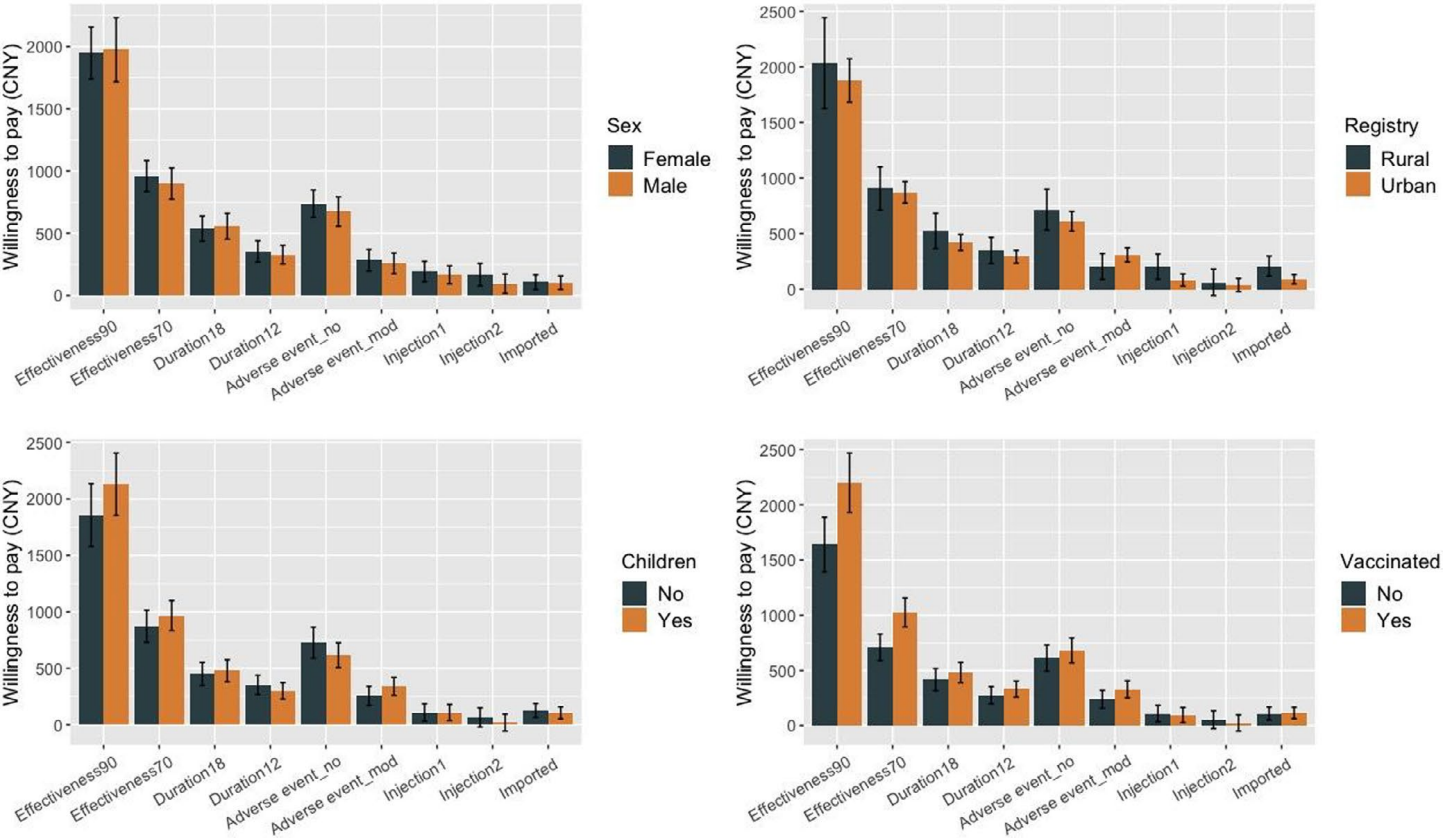
Willingness to pay estimation for subgroup population

Results of the WTP estimation supported the comparisons of the respondents’ preferences from the monetary perspective. The results demonstrated that respondents prefer to pay more for effectiveness and longer protective duration than for the other attributes. On average, respondents were willing to pay around 1948 CNY and 446 CNY to take vaccines with 90% effectiveness and a protective duration of 18 months compared with 50% effectiveness and a protective duration of six months, respectively. In terms of the frequency of injections, respondents were willing to pay only 98 CNY to take one shot rather than take three shots. Table 4 and Figure 3 present results of the selective subgroup analysis. The COVID‐19 vaccine with higher effectiveness was more likely to lead to a higher utility value for respondents who were women, lived in a rural area, parenting children and had vaccinated experience. The utility values and probability of selection for all design profiles are presented in the Appendix (Table A2).

**TABLE 4:** Results of subgroup analysis (n = 1236)

	Male		Female	
	Coefficient (SE)	SD (SE)	Coefficient (SE)	SD (SE)
Effect 70%	1.486 (0.076)***	0.593 (0.111)***	1.544 (0.073)***	0.225 (0.184)
Effect 90%	3.279 (0.147)***	1.954 (0.129)***	3.391 (0.149)***	0.265 (0.187)***
Duration 12 mo	0.464 (0.06)***	0.011 (0.101)	0.564 (0.059)***	1.866 (0.131)
Duration 18 mo	0.601 (0.072)***	0.351 (0.135)*	0.955 (0.079)***	0.081 (0.116)***
Moderate adverse event	0.524 (0.062)***	0.075 (0.136)	0.444 (0.07)***	0.6 (0.123)***
No adverse event	0.951 (0.079)***	0.93 (0.096)**	1.157 (0.085)***	0.71 (0.1)***
Injection 2 times	−0.04 (0.065)	0.319 (0.137)	0.164 (0.066)***	1.156 (0.097)
Injection 1 time	0.076 (0.059)	0.066 (0.158)	0.286 (0.062)*	0.083 (0.115)*
Imported	0.198 (0.045)***	0.085 (0.173)	0.176 (0.046)***	0.317 (0.127)
Price	−0.001 (0.001)***	0.002 (0.001)***	−0.002 (0.001)***	0.213 (0.113)***

	Urban resident		Rural resident	
	Coefficient (SE)	SD (SE)	Coefficient (SE)	SD (SE)
Effect 70%	1.488 (0.058)***	0.372 (0.102)***	1.44 (0.11)***	0.564 (0.191)**
Effect 90%	3.235 (0.109)***	1.805 (0.101)***	3.208 (0.227)***	2.087 (0.233)***
Duration 12 mo	0.499 (0.047)***	0.041 (0.086)	0.554 (0.089)***	0.1 (0.148)
Duration 18 mo	0.718 (0.056)***	0.326 (0.122)**	0.832 (0.118)**	0.683 (0.182)***
Moderate adverse event	0.528 (0.052)***	0.423 (0.096)***	0.324 (0.093)***	0.196 (0.24)
No adverse event	1.043 (0.064)***	0.933 (0.078)***	1.137 (0.126)***	0.968 (0.176)***
Injection 2 times	0.065 (0.051)	0.02 (0.148)	0.099 (0.095)	0.07 (0.178)
Injection 1 time	0.142 (0.048)**	0.144 (0.128)	0.323 (0.09)***	0.309 (0.202)
Imported	0.154 (0.036)***	0.299 (0.07)***	0.331 (0.068)***	0.192 (0.141)
Price	−0.002 (0.001)***	0.002 (0)***	−0.002 (0.001)***	0.002 (0.001)***

	Had children		No children	
	Coefficient (SE)	SD (SE)	Coefficient (SE)	SD (SE)
Effect 70%	1.558 (0.071)***	0.448 (0.126)***	1.411 (0.081)***	0.517 (0.127)***
Effect 90%	3.426 (0.139)***	2.015 (0.138)***	3.001 (0.141)***	1.782 (0.137)***
Duration 12 months	0.483 (0.057)***	0.081 (0.098)	0.57 (0.065)***	0.123 (0.118)
Duration 18 months	0.77 (0.072)***	0.606 (0.113)***	0.727 (0.076)***	0.292 (0.215)
Moderate adverse event	0.546 (0.06)***	0.072 (0.155)	0.413 (0.067)***	0.211 (0.295)
No adverse event	0.991 (0.076)***	0.947 (0.092)***	1.176 (0.094)***	1.092 (0.101)***
Injection 2 times	0.031 (0.062)	0.126 (0.129)	0.105 (0.069)	0.27 (0.145)
Injection 1 time	0.175 (0.058)*	0.336 (0.118)**	0.176 (0.063)*	0.115 (0.248)
Imported	0.169 (0.043)***	0.286 (0.102)**	0.204 (0.049)***	0.326 (0.088)***
Price	−0.002 (0.001)***	0.002 (0.001)***	−0.002 (0.001)***	0.002 (0.001)***

	Vaccinated		Non‐vaccinated	
	Coefficient (SE)	SD (SE)	Coefficient (SE)	SD (SE)
Effect 70%	1.601 (0.065)***	0.212 (0.157)	1.259 (0.079)***	0.584 (0.128)***
Effect 90%	3.434 (0.129)***	1.911 (0.119)	2.912 (0.151)***	1.809 (0.145)***
Duration 12 months	0.515 (0.053)***	0.116 (0.108)	0.487 (0.066)***	0.051 (0.118)
Duration 18 months	0.751 (0.066)***	0.392 (0.142)	0.74 (0.081)***	0.473 (0.128)***
Moderate adverse event	0.512 (0.057)***	0.22 (0.167)	0.423 (0.071)***	0.192 (0.133)
No adverse event	1.064 (0.077)***	1.058 (0.088)	1.087 (0.088)***	0.905 (0.107)***
Injection 2 times	0.037 (0.058)	0.211 (0.158)	0.094 (0.072)	0.091 (0.132)
Injection 1 time	0.149 (0.054)*	0.222 (0.151)	0.193 (0.067)*	0.228 (0.133)
Imported	0.179 (0.041)***	0.175 (0.094)	0.194 (0.053)***	0.45 (0.084)***
Price	−0.002 (0.001)***	0.002 (0.001)	−0.002 (0.001)***	0.002 (0.001)***

Abbreviations: SE, standard error; SD standard deviation.

* <0.05;

** <0.01;

*** <0.001.

## 4 DISCUSSION

This study reports the results of a DCE study quantifying the general public's stated preference for the COVID‐19 vaccination programme. To our knowledge, this is the first study to investigate the public's preference for selecting such vaccination programmes in China and worldwide. Results of the DCE study showed that the respondents’ vaccination probability increased with an increase in the vaccine's effectiveness and protective duration as well as with a decrease in the severity of adverse events and price. The MXL estimates further suggest the existence of preference heterogeneity in five out of six attributes.

We contribute to the existing literature by finding that the Chinese population showed higher preference for an imported rather than a domestically manufactured COVID‐19 vaccine product. This is not a surprising result, as some previous studies have indicated the Chinese people's high preference for imported vaccine.^35^, ^36^ In the past few years, China has had several vaccine‐related scandals that severely diminished the general public's trust in the quality and effectiveness of domestically manufactured health products.^22^, ^37^ Moreover, several public health scandals have recently raised concerns about the government's protectionist policy against foreign imports of vaccines. Confidence in domestic medical product manufacturers and distributors reached a new low in 2018 after a major manufacturer was found to be selling faulty rabies and 'diphtheria, tetanus and pertussis' shots, which were supposed to save lives and protect infants.^38^ Our findings confirmed that despite some Chinese pharmaceutical companies now taking a leading position in the race to develop the COVID‐19 vaccine, the long‐term vaccine crisis has had a significantly negative influence on the public's willingness to select a domestic vaccine. This is in line with the findings of previous studies that anxiety about vaccine safety reduces and even eliminates public's willingness towards taking vaccination.^24^, ^39^, ^40^ However, we found that there appeared to be trade‐offs between attributes that participants considered to maximize the vaccines’ utility. For example, when the other conditions were unchanged, a domestic vaccine that was priced lower could result in a higher probability to be selected than an imported vaccine.

The public's WTP for a COVID‐19 vaccine is rarely reported; only a recent study indicated that the Chilean public's WTP for a COVID‐19 vaccine is nearly 184 USD.^11^ In our study, both the main effect model and subgroup analyses confirmed that price has a limited influence on the public's preference for selecting a vaccine, and the highest price contributed only little to the decrease in the individuals’ overall utility. Although the leaders of several countries have already promised that the future COVID‐19 vaccines will be provided as 'public goods' and that their development will be paid for with taxes, we still include the price parameter in our DCE study. This is because, first, the way to translate these political statements into a concrete plan to provide a vaccine without charge to the public is yet to be determined. Moreover, governments of some countries, such as the United States, have confirmed that the COVID‐19 vaccine would have an actual price tag, which would limit its availability for many Americans.^41^ The second reason is related to the development of the coronavirus. A new study has confirmed that mutations can make the SARS‐CoV‐2 virus more infectious.^42^ If this is true, development of the COVID‐19 vaccine would not be a one‐off effort, but a long‐term process. The cost of providing a free COVID‐19 vaccine to the public season by season would then be a significant financial burden and an impossible mission for some developing countries. Therefore, our WTP estimations provide useful information for policymakers to develop a reasonable pricing strategy to commoditize the COVID‐19 vaccine in the market. In addition, we should not neglect the effect of 'free‐riding' behaviours, which were reported by previous studies about vaccination decisions.^16^, ^43^, ^44^ Price is likely to only have a slight influence on individuals’ vaccination preference, not because they do not care about the cost of vaccination but because they would not get vaccinated and hope to be covered by herd immunity. Herd immunity is developed when other people take the vaccine and create a sufficiently high coverage to protect everyone. Further, the price of vaccination in our study was limited to five levels, and the public's decisions on the choices might be affected by this predefined price range. However, at the time of conducting the study, no COVID‐19 vaccine was available in the market. The price range was informed by (1) the prices of the other vaccines, such as influenza and pneumonia, that are available in China, and (2) suggestions from experts who had knowledge and experience in vaccine pricing and procurement. Yet, the effect of different price range on the public's preference over vaccines should be further investigated in follow‐up studies.

The subgroup analysis further demonstrated that the female respondents were more likely to select a COVID‐19 vaccine with higher effectiveness, longer protective duration, fewer adverse events and fewer injections than the male respondents. However, the females’ preference for vaccination seemed to be more sensitive to increased price. Although previous studies indicated that females are more likely to take up other vaccines than males,^45^, ^46^ none discussed the effect of price on the decision making between males and females. Some possible explanations for this might be differences in the SES and health status, and provider bias.

This study showed that urban residents preferred a vaccine with higher effectiveness, whereas rural residents preferred longer protective duration. Although several previous studies have reported low vaccination coverage in rural populations,^47^, ^48^, ^49^ none compared the individual vaccination preferences between the rural and the urban areas. The distribution of high‐quality health‐care resources is highly uneven in China.^50^ Regarding the COVID‐19 vaccine, urban residents in this study preferred a product with higher efficiency, indicating that they are able and confident about affording another shot when the protective duration expires. However, for rural residents, health‐care systems often struggle to meet their needs.^51^ Compared with urban residents, the limited selection for rural residents makes them prefer a vaccine without very high efficiency but with a longer protective duration to reduce the frequency of visits and costs. Our findings indicate that although urban and rural people's preference to uptake a vaccine is similar to some extent, as previous studies have revealed, the main determinant of the vaccination choice remains different since high‐quality health‐care resources are perceived to be more difficult to approach in rural areas.^46^, ^52^, ^53^

Methodologically, in this study, we chose to use DCE over another stated preference method—contingent valuation (CV)—for three reasons. First, DCE provides more information than CV and allows the estimation of the marginal WTP for different levels and attributes.^54^ Second, unlike CV which directly elicits the monetary value of a product, DCE mitigates certain ethical concerns in survey research.^55^ Third, compared with CV, DCE provides better opportunities for researchers to identify people's trade‐offs between different attributes of a product.^56^ However, it is worth noting that DCE usually generates a higher cognitive burden than CV, especially when the design of a DCE is complicated or the sample size is a relatively small one.

## 5 LIMITATIONS OF THE STUDY

Several limitations of our research must be addressed. First, our data were collected from an online survey, which means that people who did not have access to the Internet were excluded from the survey, which is likely to lead to a selection bias. Second, compared with the Chinese general population, our sample is much younger, better educated and has a higher income. Nearly 80% of them reported having an average monthly income greater than the national median. Methodologically, an inherent characteristic of DCE is that respondents have to make a choice between two hypothetical profiles. However, in the real world, they might be presented with more options. Hence, the generalizability of our findings is limited. Third, the low utility of adverse events in our study might be resulted from setting up the range of adverse events at relatively milder levels in the first place. Furthermore, although explanations on the attributes and levels of the profiles were provided in the survey, some participants might not read them carefully or even misunderstood the profiles. Therefore, the validity of our findings is not without concern. Finally, a more heterogeneous approach is needed in future studies by including different stated preference methods such as CV, or statistical techniques such as hierarchical Bayes.

## 6 CONCLUSIONS

This study found that 80% of the Chinese public who participated in the survey preferred to receive the COVID‐19 vaccination when it is available. More than 40% of them indicated that the elderly should be prioritized for the vaccination programme. When the participants were facing trade‐offs between two COVID‐19 vaccination programmes, effectiveness was regarded as the most important attribute, followed by long protective duration, very few adverse events and being manufactured overseas. Interestingly, price was the least important attribute affecting the public preference in selecting the COVID‐19 vaccine.

However, such findings need to be interpreted with caution. The distribution of income levels among our sample was skewed towards the higher end of national average. The public with lower incomes who will be more sensitive to prices was in fact unrepresented. Moreover, since the SARS‐CoV‐2 is still mutating, it is hard to predict the effectiveness of the vaccines that are currently under development, and thus, the final prices of these vaccines are largely unknown. Therefore, we suggest that price should not be considered as less important when the industry and the government design and implement marketing and policy strategies related to the COVID‐19 vaccines. It is also worth noting that different population subgroups had heterogeneous or varied preferences on the vaccine, which further reminds us of the importance of taking individuals’ or a certain social group's needs into consideration for any vaccination programme. Follow‐up studies from other countries are needed to investigate how the public's acceptance and preference for COVID‐19 vaccination change over time as the pandemic progresses. Not only does the development of vaccines against COVID‐19 has to be a global effort, building trust in and promoting equity access to the COVID‐19 vaccines also require co‐operation at the global level.

## CONFLICT OF INTEREST

All the authors declare no conflict of interest.

## AUTHOR CONTRIBUTIONS

DD, RHX, DF and SW conceptualized and designed the research protocol of the study. EW, CH, EY and FZC commented on the research design and helped revise the design. DD and RHX implemented data collection and interpretation. DD and RHX wrote the first draft of the manuscript. CH ad SW revised the first draft. All authors were involved in revising the article in the third round and approved the final manuscript.

## ETHICAL APPROVAL

The study was approved by the Survey and Behavioural Research Ethics Committee of the Chinese University of Hong Kong. The ethics approval code is SBRE‐19‐690.

## 7 APPENDIX

7.1

**TABLE A1:** All participants’ characteristics for each block (n = 1277)

	Block 1 (n = 322)		Block 2 (n = 313)		Block 3 (n = 322)		Block 4 (n = 320)	
	N	%	n	%	n	%	n	%
Sex								
Male	171	53.1	152	48.6	164	50.9	139	43.4
Female	151	46.9	161	51.4	158	49.1	181	56.6
Age, mean (SD)	28.9	7.5	30.2	7.5	31.0	8.6	30.0	6.8
Educational level								
Secondary and below	62	17.4	60	19.2	53	26.6	58	18.2
Tertiary and above	260	82.6	253	80.8	269	83.4	262	81.8
Marital status								
Unmarried	137	42.6	126	40.3	116	36	116	36.3
Married	183	56.8	185	59.1	205	63.7	202	61.1
Divorced	2	0.6	2	0.6	1	0.3	2	0.6
Family register								
Urban area	246	76.4	228	72.8	264	82	248	77.5
Rural area	75	23.3	85	27.2	58	18	72	22.5
Number of children								
0	154	47.8	142	45.4	131	40.7	129	40.3
1	131	40.7	145	46.3	166	51.6	158	49.4
≥2	37	11.5	26	8.3	25	7.7	33	10.3
Living status								
Live along	33	10.3	23	7.4	22	6.8	28	8.7
Live with family	263	81.7	272	87	285	88.5	227	88.6
Live with friends	22	6.8	18	5.6	12	3.7	15	4.7
Others	4	1.2	‐	‐	3	0.9	‐	‐
Working status								
Full‐time employed	249	77.3	254	81.2	250	77.6	245	76.6
Part‐time employed	12	3.7	11	3.5	10	3.1	14	4.4
Farming	2	0.6	3	0.9	3	0.9	2	0.6
Students	53	16.5	41	13.1	57	17.7	51	15.9
Housewife	1	0.3	2	0.6	‐	‐	1	0.3
Retired	2	0.6	‐	‐	1	0.3	1	0.3
Unemployed	3	0.9	2	0.6	1	0.3	6	1.9
Medical insurance								
Yes	317	98.4	306	97.8	319	99.1	315	98.4
No	5	1.6	7	2.2	3	0.9	5	1.6
Personal income (CNY/month)								
<1000	23	7.1	25	7.9	29	9	23	7.2
1000‐1999	24	7.5	21	6.7	21	6.5	23	7.2
2000‐2999	25	7.8	17	5.4	9	2.8	24	7.5
3000‐3999	28	8.7	32	10.2	31	9.6	24	7.5
4000‐4999	20	6.2	21	6.7	18	5.6	28	8.7
5000‐5999	34	10.6	35	11.2	35	10.8	33	10.3
6000‐6999	32	9.9	34	10.9	32	9.9	29	9.1
7000‐7999	24	7.5	31	9.9	22	6.8	28	8.7
8000‐8999	35	10.9	30	9.6	36	11.2	40	12.5
9000‐9999	19	5.9	27	8.6	19	5.9	27	8.4
≥10 000	58	18.0	40	12.8	70	21.7	41	12.8

Note

1277 including the participants who correctly answered the trap question, but indicated the discrete choice experiment questions are hard to be understood.

**TABLE A2:** Utility score of all the profiles in this study design

No.	Effect	Duration	Adverse	Injection	Cost	Place	Utility	Percentage (%)
1	90	18	No	1	400	Imported	8.083	1.128
2	90	18	No	3	400	Imported	8.083	1.128
3	90	18	No	1	600	Imported	8.081	1.126
4	90	18	No	2	1000	Imported	8.077	1.122
5	90	18	No	1	0	Domestic	7.909	0.948
6	90	18	No	1	200	Domestic	7.907	0.946
7	90	18	No	2	200	Domestic	7.907	0.946
8	90	18	No	2	400	Domestic	7.905	0.944
9	90	18	No	2	800	Domestic	7.901	0.941
10	90	18	No	1	1000	Domestic	7.899	0.939
11	90	18	No	3	1000	Domestic	7.899	0.939
12	90	12	No	3	200	Imported	7.857	0.9
13	90	12	No	1	400	Imported	7.855	0.898
14	90	12	No	2	400	Imported	7.855	0.898
15	90	12	No	2	600	Imported	7.853	0.896
16	90	12	No	1	800	Imported	7.851	0.895
17	90	12	No	3	800	Imported	7.851	0.895
18	90	12	No	1	1000	Imported	7.849	0.893
19	90	12	No	3	1000	Imported	7.849	0.893
20	90	12	No	1	0	Domestic	7.681	0.755
21	90	12	No	2	0	Domestic	7.681	0.755
22	90	12	No	1	200	Domestic	7.679	0.753
23	90	12	No	2	200	Domestic	7.679	0.753
24	90	12	No	3	400	Domestic	7.677	0.752
25	90	12	No	3	600	Domestic	7.675	0.75
26	90	12	No	2	1000	Domestic	7.671	0.747
27	90	18	Moderate	3	0	Imported	7.516	0.64
28	90	18	Moderate	3	200	Imported	7.514	0.639
29	90	18	Moderate	2	800	Imported	7.508	0.635
30	90	18	Moderate	3	800	Imported	7.508	0.635
31	90	18	Moderate	3	1000	Imported	7.506	0.634
32	90	6	No	1	400	Imported	7.364	0.55
33	90	6	No	3	800	Imported	7.36	0.548
34	90	18	Moderate	1	0	Domestic	7.338	0.536
35	90	18	Moderate	2	0	Domestic	7.338	0.536
36	90	18	Moderate	2	200	Domestic	7.336	0.535
37	90	18	Moderate	1	400	Domestic	7.334	0.534
38	90	18	Moderate	2	400	Domestic	7.334	0.534
39	90	18	Moderate	3	400	Domestic	7.334	0.534
40	90	18	Moderate	2	600	Domestic	7.332	0.532
41	90	18	Moderate	3	600	Domestic	7.332	0.532
42	90	18	Moderate	1	1000	Domestic	7.328	0.53
43	90	12	Moderate	2	200	Imported	7.286	0.509
44	90	12	Moderate	2	800	Imported	7.28	0.505
45	90	6	No	2	0	Domestic	7.19	0.462
46	90	6	No	3	0	Domestic	7.19	0.462
47	90	6	No	3	200	Domestic	7.188	0.461
48	90	6	No	2	400	Domestic	7.186	0.46
49	90	6	No	3	400	Domestic	7.186	0.46
50	90	6	No	3	600	Domestic	7.184	0.459
51	90	6	No	1	1000	Domestic	7.18	0.457
52	90	6	No	2	1000	Domestic	7.18	0.457
53	90	12	Moderate	2	0	Domestic	7.11	0.426
54	90	12	Moderate	1	200	Domestic	7.108	0.426
55	90	12	Moderate	3	200	Domestic	7.108	0.426
56	90	12	Moderate	3	400	Domestic	7.106	0.425
57	90	12	Moderate	1	600	Domestic	7.104	0.424
58	90	12	Moderate	1	800	Domestic	7.102	0.423
59	90	12	Moderate	3	800	Domestic	7.102	0.423
60	90	18	No	1	0	Imported	7.087	0.417
61	90	18	No	3	0	Imported	7.087	0.417
62	90	18	No	1	200	Imported	7.085	0.416
63	90	18	No	3	200	Imported	7.085	0.416
64	90	18	No	2	400	Imported	7.083	0.415
65	90	18	No	1	800	Imported	7.079	0.413
66	90	18	No	3	800	Imported	7.079	0.413
67	90	18	Severe	2	0	Imported	7.045	0.4
68	90	18	Severe	2	200	Imported	7.043	0.399
69	90	18	Severe	1	600	Imported	7.039	0.397
70	90	18	Severe	3	600	Imported	7.039	0.397
71	90	18	No	2	0	Domestic	6.909	0.349
72	90	18	No	1	400	Domestic	6.905	0.347
73	90	18	No	3	400	Domestic	6.905	0.347
74	90	18	No	2	600	Domestic	6.903	0.347
75	90	18	No	3	600	Domestic	6.903	0.347
76	90	18	Severe	3	0	Domestic	6.867	0.334
77	90	18	Severe	1	200	Domestic	6.865	0.334
78	90	18	Severe	3	400	Domestic	6.863	0.333
79	90	18	Severe	2	600	Domestic	6.861	0.332
80	90	12	No	1	0	Imported	6.859	0.332
81	90	12	No	2	0	Imported	6.859	0.332
82	90	12	No	3	0	Imported	6.859	0.332
83	90	18	Severe	1	800	Domestic	6.859	0.332
84	90	12	No	2	200	Imported	6.857	0.331
85	90	18	Severe	1	1000	Domestic	6.857	0.331
86	90	18	Severe	2	1000	Domestic	6.857	0.331
87	90	12	No	3	400	Imported	6.855	0.33
88	90	12	No	3	600	Imported	6.853	0.33
89	90	12	No	2	1000	Imported	6.849	0.328
90	90	12	Severe	2	0	Imported	6.817	0.318
91	90	12	Severe	3	0	Imported	6.817	0.318
92	90	12	Severe	3	200	Imported	6.815	0.318
93	90	12	Severe	2	400	Imported	6.813	0.317
94	90	12	Severe	2	600	Imported	6.811	0.316
95	90	12	Severe	3	1000	Imported	6.807	0.315
96	90	6	Moderate	1	0	Imported	6.797	0.312
97	90	6	Moderate	3	0	Imported	6.797	0.312
98	90	6	Moderate	1	200	Imported	6.795	0.311
99	90	6	Moderate	3	200	Imported	6.795	0.311
100	90	6	Moderate	2	600	Imported	6.791	0.31
101	90	6	Moderate	1	800	Imported	6.789	0.309
102	90	6	Moderate	1	1000	Imported	6.787	0.309
103	90	12	No	1	400	Domestic	6.677	0.277
104	90	12	No	2	400	Domestic	6.677	0.277
105	90	12	No	1	600	Domestic	6.675	0.276
106	90	12	No	1	800	Domestic	6.673	0.275
107	90	12	No	2	800	Domestic	6.673	0.275
108	90	12	No	3	800	Domestic	6.673	0.275
109	90	12	No	1	1000	Domestic	6.671	0.275
110	90	12	Severe	1	200	Domestic	6.637	0.266
111	90	12	Severe	1	400	Domestic	6.635	0.265
112	90	12	Severe	1	600	Domestic	6.633	0.265
113	90	12	Severe	3	600	Domestic	6.633	0.265
114	90	12	Severe	1	800	Domestic	6.631	0.264
115	90	12	Severe	2	800	Domestic	6.631	0.264
116	90	12	Severe	1	1000	Domestic	6.629	0.264
117	90	12	Severe	2	1000	Domestic	6.629	0.264
118	90	6	Moderate	2	200	Domestic	6.617	0.26
119	90	6	Moderate	1	400	Domestic	6.615	0.26
120	90	6	Moderate	1	600	Domestic	6.613	0.259
121	90	6	Moderate	3	600	Domestic	6.613	0.259
122	90	6	Moderate	2	800	Domestic	6.611	0.259
123	90	6	Moderate	2	1000	Domestic	6.609	0.258
124	90	6	Moderate	3	1000	Domestic	6.609	0.258
125	90	18	Moderate	2	200	Imported	6.514	0.235
126	90	18	Moderate	2	400	Imported	6.512	0.235
127	90	18	Moderate	3	400	Imported	6.512	0.235
128	90	18	Moderate	3	600	Imported	6.51	0.234
129	90	18	Moderate	1	800	Imported	6.508	0.234
130	90	6	No	1	0	Imported	6.368	0.203
131	90	6	No	1	200	Imported	6.366	0.203
132	90	6	No	3	200	Imported	6.366	0.203
133	90	6	No	3	400	Imported	6.364	0.202
134	70	18	No	1	200	Imported	6.363	0.202
135	70	18	No	3	200	Imported	6.363	0.202
136	90	6	No	1	800	Imported	6.36	0.201
137	90	6	No	2	800	Imported	6.36	0.201
138	70	18	No	1	800	Imported	6.357	0.201
139	70	18	No	3	800	Imported	6.357	0.201
140	70	18	No	1	1000	Imported	6.355	0.2
141	90	18	Moderate	3	0	Domestic	6.338	0.197
142	90	18	Moderate	1	200	Domestic	6.336	0.197
143	90	18	Moderate	3	200	Domestic	6.336	0.197
144	90	18	Moderate	1	600	Domestic	6.332	0.196
145	90	18	Moderate	2	800	Domestic	6.33	0.195
146	90	18	Moderate	3	800	Domestic	6.33	0.195
147	90	18	Moderate	2	1000	Domestic	6.328	0.195
148	90	6	Severe	2	0	Imported	6.326	0.195
149	90	6	Severe	1	600	Imported	6.32	0.194
150	90	12	Moderate	1	0	Imported	6.288	0.187
151	90	12	Moderate	3	0	Imported	6.288	0.187
152	90	12	Moderate	2	400	Imported	6.284	0.187
153	90	12	Moderate	2	600	Imported	6.282	0.186
154	90	12	Moderate	1	800	Imported	6.28	0.186
155	90	6	No	2	200	Domestic	6.188	0.17
156	70	18	No	3	0	Domestic	6.187	0.169
157	90	6	No	1	400	Domestic	6.186	0.169
158	70	18	No	2	200	Domestic	6.185	0.169
159	90	6	No	1	600	Domestic	6.184	0.169
160	90	6	No	2	600	Domestic	6.184	0.169
161	70	18	No	1	400	Domestic	6.183	0.169
162	90	6	No	3	800	Domestic	6.182	0.169
163	70	18	No	1	600	Domestic	6.181	0.168
164	70	18	No	2	600	Domestic	6.181	0.168
165	90	6	No	3	1000	Domestic	6.18	0.168
166	70	18	No	2	800	Domestic	6.179	0.168
167	90	6	Severe	1	0	Domestic	6.148	0.163
168	90	6	Severe	3	0	Domestic	6.148	0.163
169	90	6	Severe	1	400	Domestic	6.144	0.162
170	90	6	Severe	2	600	Domestic	6.142	0.162
171	90	6	Severe	3	600	Domestic	6.142	0.162
172	90	6	Severe	2	800	Domestic	6.14	0.162
173	90	6	Severe	3	800	Domestic	6.14	0.162
174	90	6	Severe	3	1000	Domestic	6.138	0.161
175	70	12	No	2	0	Imported	6.137	0.161
176	70	12	No	2	200	Imported	6.135	0.161
177	70	12	No	3	600	Imported	6.131	0.16
178	70	12	No	1	800	Imported	6.129	0.16
179	70	12	No	2	800	Imported	6.129	0.16
180	70	12	No	2	1000	Imported	6.127	0.16
181	90	12	Moderate	1	400	Domestic	6.106	0.156
182	90	12	Moderate	3	600	Domestic	6.104	0.156
183	90	12	Moderate	2	800	Domestic	6.102	0.156
184	90	12	Moderate	1	1000	Domestic	6.1	0.155
185	90	12	Moderate	2	1000	Domestic	6.1	0.155
186	90	12	Moderate	3	1000	Domestic	6.1	0.155
187	90	18	No	2	0	Imported	6.087	0.153
188	90	18	No	2	200	Imported	6.085	0.153
189	90	18	No	2	600	Imported	6.081	0.152
190	90	18	No	3	600	Imported	6.081	0.152
191	90	18	No	2	800	Imported	6.079	0.152
192	90	18	No	1	1000	Imported	6.077	0.152
193	90	18	No	3	1000	Imported	6.077	0.152
194	90	18	Severe	3	0	Imported	6.045	0.147
195	90	18	Severe	1	200	Imported	6.043	0.147
196	90	18	Severe	1	400	Imported	6.041	0.146
197	90	18	Severe	2	400	Imported	6.041	0.146
198	90	18	Severe	1	800	Imported	6.037	0.146
199	90	18	Severe	3	800	Imported	6.037	0.146
200	90	18	Severe	1	1000	Imported	6.035	0.146
201	90	18	Severe	3	1000	Imported	6.035	0.146
202	70	12	No	1	0	Domestic	5.959	0.135
203	70	12	No	1	200	Domestic	5.957	0.135
204	70	12	No	3	200	Domestic	5.957	0.135
205	70	12	No	1	400	Domestic	5.955	0.134
206	70	12	No	2	400	Domestic	5.955	0.134
207	70	12	No	1	600	Domestic	5.953	0.134
208	70	12	No	2	600	Domestic	5.953	0.134
209	70	12	No	3	800	Domestic	5.951	0.134
210	70	12	No	3	1000	Domestic	5.949	0.134
211	90	18	No	3	0	Domestic	5.909	0.128
212	90	18	No	3	200	Domestic	5.907	0.128
213	90	18	No	1	600	Domestic	5.903	0.128
214	90	18	No	1	800	Domestic	5.901	0.127
215	90	18	No	3	800	Domestic	5.901	0.127
216	90	18	No	2	1000	Domestic	5.899	0.127
217	90	18	Severe	1	0	Domestic	5.867	0.123
218	90	18	Severe	2	0	Domestic	5.867	0.123
219	90	18	Severe	3	200	Domestic	5.865	0.123
220	90	18	Severe	2	800	Domestic	5.859	0.122
221	90	12	No	1	200	Imported	5.857	0.122
222	90	12	No	1	600	Imported	5.853	0.121
223	90	12	No	2	800	Imported	5.851	0.121
224	90	12	Severe	1	0	Imported	5.817	0.117
225	90	12	Severe	1	600	Imported	5.811	0.116
226	90	12	Severe	3	600	Imported	5.811	0.116
227	90	12	Severe	1	800	Imported	5.809	0.116
228	90	12	Severe	2	800	Imported	5.809	0.116
229	90	12	Severe	3	800	Imported	5.809	0.116
230	90	12	Severe	2	1000	Imported	5.807	0.116
231	90	6	Moderate	2	200	Imported	5.795	0.114
232	70	18	Moderate	3	0	Imported	5.794	0.114
233	90	6	Moderate	1	400	Imported	5.793	0.114
234	90	6	Moderate	2	400	Imported	5.793	0.114
235	90	6	Moderate	3	400	Imported	5.793	0.114
236	90	6	Moderate	1	600	Imported	5.791	0.114
237	70	18	Moderate	3	400	Imported	5.79	0.114
238	90	6	Moderate	2	800	Imported	5.789	0.114
239	70	18	Moderate	3	600	Imported	5.788	0.114
240	90	6	Moderate	2	1000	Imported	5.787	0.114
241	90	12	No	3	0	Domestic	5.681	0.102
242	90	12	No	3	200	Domestic	5.679	0.102
243	90	12	No	2	600	Domestic	5.675	0.102
244	90	12	No	3	1000	Domestic	5.671	0.101
245	70	6	No	1	200	Imported	5.644	0.098
246	90	12	Severe	2	0	Domestic	5.639	0.098
247	90	12	Severe	3	0	Domestic	5.639	0.098
248	70	6	No	1	800	Imported	5.638	0.098
249	90	12	Severe	2	200	Domestic	5.637	0.098
250	90	12	Severe	2	400	Domestic	5.635	0.098
251	90	12	Severe	3	400	Domestic	5.635	0.098
252	90	12	Severe	2	600	Domestic	5.633	0.097
253	90	6	Moderate	1	0	Domestic	5.619	0.096
254	90	6	Moderate	2	0	Domestic	5.619	0.096
255	90	6	Moderate	3	0	Domestic	5.619	0.096
256	90	6	Moderate	1	200	Domestic	5.617	0.096
257	70	18	Moderate	1	200	Domestic	5.614	0.096
258	70	18	Moderate	2	200	Domestic	5.614	0.096
259	70	18	Moderate	1	400	Domestic	5.612	0.095
260	70	18	Moderate	2	400	Domestic	5.612	0.095
261	90	6	Moderate	1	800	Domestic	5.611	0.095
262	90	6	Moderate	3	800	Domestic	5.611	0.095
263	70	18	Moderate	2	600	Domestic	5.61	0.095
264	70	18	Moderate	1	800	Domestic	5.608	0.095
265	70	18	Moderate	2	800	Domestic	5.608	0.095
266	70	18	Moderate	3	800	Domestic	5.608	0.095
267	70	18	Moderate	2	1000	Domestic	5.606	0.095
268	70	18	Moderate	3	1000	Domestic	5.606	0.095
269	70	12	Moderate	1	0	Imported	5.566	0.091
270	70	12	Moderate	3	0	Imported	5.566	0.091
271	70	12	Moderate	1	200	Imported	5.564	0.091
272	70	12	Moderate	2	600	Imported	5.56	0.091
273	90	18	Moderate	1	0	Imported	5.516	0.087
274	90	18	Moderate	2	0	Imported	5.516	0.087
275	90	18	Moderate	1	200	Imported	5.514	0.086
276	90	18	Moderate	1	400	Imported	5.512	0.086
277	90	18	Moderate	1	600	Imported	5.51	0.086
278	90	18	Moderate	2	600	Imported	5.51	0.086
279	90	18	Moderate	1	1000	Imported	5.506	0.086
280	90	18	Moderate	2	1000	Imported	5.506	0.086
281	70	6	No	1	0	Domestic	5.468	0.083
282	70	6	No	2	200	Domestic	5.466	0.082
283	70	6	No	3	200	Domestic	5.466	0.082
284	70	6	No	2	400	Domestic	5.464	0.082
285	70	6	No	3	400	Domestic	5.464	0.082
286	70	6	No	3	600	Domestic	5.462	0.082
287	70	6	No	2	800	Domestic	5.46	0.082
288	70	6	No	3	800	Domestic	5.46	0.082
289	70	6	No	3	1000	Domestic	5.458	0.082
290	70	12	Moderate	2	0	Domestic	5.388	0.076
291	70	12	Moderate	2	400	Domestic	5.384	0.076
292	70	12	Moderate	1	600	Domestic	5.382	0.076
293	70	12	Moderate	3	600	Domestic	5.382	0.076
294	70	12	Moderate	3	800	Domestic	5.38	0.076
295	70	12	Moderate	1	1000	Domestic	5.378	0.075
296	90	6	No	2	0	Imported	5.368	0.075
297	90	6	No	3	0	Imported	5.368	0.075
298	90	6	No	2	200	Imported	5.366	0.075
299	70	18	No	1	0	Imported	5.365	0.074
300	70	18	No	2	0	Imported	5.365	0.074
301	90	6	No	2	400	Imported	5.364	0.074
302	90	6	No	1	600	Imported	5.362	0.074
303	90	6	No	2	600	Imported	5.362	0.074
304	90	6	No	3	600	Imported	5.362	0.074
305	70	18	No	1	400	Imported	5.361	0.074
306	70	18	No	3	400	Imported	5.361	0.074
307	70	18	No	1	600	Imported	5.359	0.074
308	70	18	No	3	600	Imported	5.359	0.074
309	90	6	No	1	1000	Imported	5.358	0.074
310	90	6	No	2	1000	Imported	5.358	0.074
311	90	6	No	3	1000	Imported	5.358	0.074
312	70	18	No	2	800	Imported	5.357	0.074
313	90	18	Moderate	1	800	Domestic	5.33	0.072
314	90	18	Moderate	3	1000	Domestic	5.328	0.072
315	90	6	Severe	1	0	Imported	5.326	0.072
316	70	18	Severe	1	0	Imported	5.323	0.071
317	90	6	Severe	2	400	Imported	5.322	0.071
318	90	6	Severe	3	400	Imported	5.322	0.071
319	70	18	Severe	1	200	Imported	5.321	0.071
320	70	18	Severe	2	400	Imported	5.319	0.071
321	90	6	Severe	1	800	Imported	5.318	0.071
322	70	18	Severe	2	600	Imported	5.317	0.071
323	90	6	Severe	1	1000	Imported	5.316	0.071
324	90	6	Severe	3	1000	Imported	5.316	0.071
325	70	18	Severe	1	1000	Imported	5.313	0.071
326	70	18	Severe	3	1000	Imported	5.313	0.071
327	90	12	Moderate	2	0	Imported	5.288	0.069
328	90	12	Moderate	1	200	Imported	5.286	0.069
329	90	12	Moderate	3	200	Imported	5.286	0.069
330	90	12	Moderate	1	400	Imported	5.284	0.069
331	90	12	Moderate	3	400	Imported	5.284	0.069
332	90	12	Moderate	1	600	Imported	5.282	0.069
333	90	12	Moderate	3	600	Imported	5.282	0.069
334	90	12	Moderate	3	800	Imported	5.28	0.068
335	90	12	Moderate	1	1000	Imported	5.278	0.068
336	90	12	Moderate	2	1000	Imported	5.278	0.068
337	90	12	Moderate	3	1000	Imported	5.278	0.068
338	90	6	No	1	0	Domestic	5.19	0.063
339	90	6	No	1	200	Domestic	5.188	0.062
340	70	18	No	2	400	Domestic	5.183	0.062
341	90	6	No	1	800	Domestic	5.182	0.062
342	90	6	No	2	800	Domestic	5.182	0.062
343	70	18	No	1	800	Domestic	5.179	0.062
344	70	18	No	3	800	Domestic	5.179	0.062
345	70	18	No	2	1000	Domestic	5.177	0.062
346	70	18	No	3	1000	Domestic	5.177	0.062
347	90	6	Severe	2	0	Domestic	5.148	0.06
348	90	6	Severe	1	200	Domestic	5.146	0.06
349	90	6	Severe	2	200	Domestic	5.146	0.06
350	90	6	Severe	3	200	Domestic	5.146	0.06
351	70	18	Severe	2	0	Domestic	5.145	0.06
352	70	18	Severe	2	200	Domestic	5.143	0.06
353	70	18	Severe	3	200	Domestic	5.143	0.06
354	70	18	Severe	3	400	Domestic	5.141	0.06
355	70	18	Severe	1	600	Domestic	5.139	0.059
356	90	6	Severe	2	1000	Domestic	5.138	0.059
357	70	18	Severe	3	800	Domestic	5.137	0.059
358	70	12	No	1	200	Imported	5.135	0.059
359	70	12	No	3	200	Imported	5.135	0.059
360	70	18	Severe	2	1000	Domestic	5.135	0.059
361	70	12	No	1	400	Imported	5.133	0.059
362	70	12	No	2	400	Imported	5.133	0.059
363	70	12	No	3	400	Imported	5.133	0.059
364	70	12	No	2	600	Imported	5.131	0.059
365	70	12	No	3	800	Imported	5.129	0.059
366	70	12	No	3	1000	Imported	5.127	0.059
367	90	12	Moderate	1	0	Domestic	5.11	0.058
368	90	12	Moderate	3	0	Domestic	5.11	0.058
369	90	12	Moderate	2	200	Domestic	5.108	0.058
370	90	12	Moderate	2	400	Domestic	5.106	0.057
371	90	12	Moderate	2	600	Domestic	5.104	0.057
372	70	12	Severe	2	400	Imported	5.091	0.057
373	70	12	Severe	3	400	Imported	5.091	0.057
374	70	12	Severe	3	600	Imported	5.089	0.057
375	70	12	Severe	2	800	Imported	5.087	0.056
376	70	12	Severe	2	1000	Imported	5.085	0.056
377	70	6	Moderate	1	0	Imported	5.075	0.056
378	70	6	Moderate	2	0	Imported	5.075	0.056
379	70	6	Moderate	2	200	Imported	5.073	0.056
380	70	6	Moderate	1	400	Imported	5.071	0.056
381	70	6	Moderate	3	400	Imported	5.071	0.056
382	70	6	Moderate	1	600	Imported	5.069	0.055
383	70	6	Moderate	3	600	Imported	5.069	0.055
384	70	6	Moderate	2	1000	Imported	5.065	0.055
385	90	18	Severe	1	0	Imported	5.045	0.054
386	90	18	Severe	3	200	Imported	5.043	0.054
387	90	18	Severe	3	400	Imported	5.041	0.054
388	90	18	Severe	2	600	Imported	5.039	0.054
389	90	18	Severe	2	800	Imported	5.037	0.054
390	90	18	Severe	2	1000	Imported	5.035	0.054
391	70	12	No	2	0	Domestic	4.959	0.05
392	70	12	No	3	0	Domestic	4.959	0.05
393	70	12	No	2	200	Domestic	4.957	0.05
394	70	12	No	1	800	Domestic	4.951	0.049
395	70	12	No	2	800	Domestic	4.951	0.049
396	50	18	No	2	0	Imported	4.949	0.049
397	70	12	No	1	1000	Domestic	4.949	0.049
398	50	18	No	2	200	Imported	4.947	0.049
399	50	18	No	1	600	Imported	4.943	0.049
400	50	18	No	3	600	Imported	4.943	0.049
401	70	12	Severe	1	0	Domestic	4.917	0.048
402	70	12	Severe	2	0	Domestic	4.917	0.048
403	70	12	Severe	3	0	Domestic	4.917	0.048
404	70	12	Severe	2	200	Domestic	4.915	0.047
405	70	12	Severe	3	200	Domestic	4.915	0.047
406	70	12	Severe	1	600	Domestic	4.911	0.047
407	70	12	Severe	1	800	Domestic	4.909	0.047
408	70	12	Severe	1	1000	Domestic	4.907	0.047
409	70	12	Severe	3	1000	Domestic	4.907	0.047
410	70	6	Moderate	3	0	Domestic	4.897	0.047
411	70	6	Moderate	3	200	Domestic	4.895	0.047
412	70	6	Moderate	2	600	Domestic	4.891	0.046
413	70	6	Moderate	1	800	Domestic	4.889	0.046
414	70	6	Moderate	1	1000	Domestic	4.887	0.046
415	70	6	Moderate	3	1000	Domestic	4.887	0.046
416	90	18	Severe	2	200	Domestic	4.865	0.045
417	90	18	Severe	1	400	Domestic	4.863	0.045
418	90	18	Severe	2	400	Domestic	4.863	0.045
419	90	18	Severe	1	600	Domestic	4.861	0.045
420	90	18	Severe	3	600	Domestic	4.861	0.045
421	90	18	Severe	3	800	Domestic	4.859	0.045
422	90	18	Severe	3	1000	Domestic	4.857	0.045
423	90	12	Severe	1	200	Imported	4.815	0.043
424	90	12	Severe	2	200	Imported	4.815	0.043
425	90	12	Severe	1	400	Imported	4.813	0.043
426	90	12	Severe	3	400	Imported	4.813	0.043
427	90	12	Severe	1	1000	Imported	4.807	0.043
428	90	6	Moderate	2	0	Imported	4.797	0.042
429	70	18	Moderate	1	0	Imported	4.794	0.042
430	70	18	Moderate	2	0	Imported	4.794	0.042
431	90	6	Moderate	3	600	Imported	4.791	0.042
432	90	6	Moderate	3	800	Imported	4.789	0.042
433	70	18	Moderate	2	600	Imported	4.788	0.042
434	90	6	Moderate	3	1000	Imported	4.787	0.042
435	70	18	Moderate	2	800	Imported	4.786	0.042
436	70	18	Moderate	3	800	Imported	4.786	0.042
437	70	18	Moderate	3	1000	Imported	4.784	0.042
438	50	18	No	3	0	Domestic	4.771	0.041
439	50	18	No	1	200	Domestic	4.769	0.041
440	50	18	No	3	400	Domestic	4.767	0.041
441	50	18	No	2	600	Domestic	4.765	0.041
442	50	18	No	1	800	Domestic	4.763	0.041
443	50	18	No	1	1000	Domestic	4.761	0.041
444	50	18	No	2	1000	Domestic	4.761	0.041
445	50	12	No	2	0	Imported	4.721	0.039
446	50	12	No	3	0	Imported	4.721	0.039
447	50	12	No	3	200	Imported	4.719	0.039
448	50	12	No	2	400	Imported	4.717	0.039
449	50	12	No	2	600	Imported	4.715	0.039
450	50	12	No	3	1000	Imported	4.711	0.039
451	70	6	No	2	0	Imported	4.646	0.036
452	70	6	No	3	0	Imported	4.646	0.036
453	70	6	No	1	400	Imported	4.642	0.036
454	70	6	No	1	600	Imported	4.64	0.036
455	70	6	No	3	600	Imported	4.64	0.036
456	90	12	Severe	1	0	Domestic	4.639	0.036
457	70	6	No	3	800	Imported	4.638	0.036
458	90	12	Severe	3	200	Domestic	4.637	0.036
459	90	12	Severe	3	800	Domestic	4.631	0.036
460	90	12	Severe	3	1000	Domestic	4.629	0.036
461	90	6	Moderate	3	200	Domestic	4.617	0.035
462	70	18	Moderate	3	0	Domestic	4.616	0.035
463	90	6	Moderate	2	400	Domestic	4.615	0.035
464	90	6	Moderate	3	400	Domestic	4.615	0.035
465	70	18	Moderate	3	200	Domestic	4.614	0.035
466	90	6	Moderate	2	600	Domestic	4.613	0.035
467	70	18	Moderate	3	400	Domestic	4.612	0.035
468	70	18	Moderate	1	600	Domestic	4.61	0.035
469	70	18	Moderate	3	600	Domestic	4.61	0.035
470	90	6	Moderate	1	1000	Domestic	4.609	0.035
471	70	18	Moderate	1	1000	Domestic	4.606	0.035
472	70	6	Severe	2	400	Imported	4.6	0.035
473	70	6	Severe	1	1000	Imported	4.594	0.034
474	70	12	Moderate	2	0	Imported	4.566	0.033
475	70	12	Moderate	1	400	Imported	4.562	0.033
476	70	12	Moderate	3	400	Imported	4.562	0.033
477	70	12	Moderate	2	800	Imported	4.558	0.033
478	70	12	Moderate	2	1000	Imported	4.556	0.033
479	50	12	No	1	200	Domestic	4.541	0.033
480	50	12	No	1	400	Domestic	4.539	0.033
481	50	12	No	1	600	Domestic	4.537	0.033
482	50	12	No	3	600	Domestic	4.537	0.033
483	50	12	No	1	800	Domestic	4.535	0.032
484	50	12	No	2	800	Domestic	4.535	0.032
485	50	12	No	1	1000	Domestic	4.533	0.032
486	50	12	No	2	1000	Domestic	4.533	0.032
487	70	6	No	2	600	Domestic	4.462	0.03
488	70	6	No	1	800	Domestic	4.46	0.03
489	70	6	No	1	1000	Domestic	4.458	0.03
490	70	6	No	2	1000	Domestic	4.458	0.03
491	70	6	Severe	3	0	Domestic	4.426	0.029
492	70	6	Severe	1	200	Domestic	4.424	0.029
493	70	6	Severe	1	400	Domestic	4.422	0.029
494	70	6	Severe	3	400	Domestic	4.422	0.029
495	70	6	Severe	1	800	Domestic	4.418	0.029
496	70	6	Severe	2	1000	Domestic	4.416	0.029
497	70	6	Severe	3	1000	Domestic	4.416	0.029
498	70	12	Moderate	1	0	Domestic	4.388	0.028
499	70	12	Moderate	3	0	Domestic	4.388	0.028
500	70	12	Moderate	2	200	Domestic	4.386	0.028
501	70	12	Moderate	3	200	Domestic	4.386	0.028
502	70	12	Moderate	1	800	Domestic	4.38	0.028
503	50	18	Moderate	1	0	Imported	4.378	0.028
504	70	12	Moderate	3	1000	Domestic	4.378	0.028
505	50	18	Moderate	3	400	Imported	4.374	0.028
506	50	18	Moderate	3	800	Imported	4.37	0.028
507	50	18	Moderate	3	1000	Imported	4.368	0.027
508	70	18	No	3	0	Imported	4.365	0.027
509	70	18	No	2	200	Imported	4.363	0.027
510	70	18	No	2	400	Imported	4.361	0.027
511	70	18	No	2	600	Imported	4.359	0.027
512	70	18	No	2	1000	Imported	4.355	0.027
513	70	18	No	3	1000	Imported	4.355	0.027
514	90	6	Severe	3	0	Imported	4.326	0.026
515	90	6	Severe	1	200	Imported	4.324	0.026
516	90	6	Severe	2	200	Imported	4.324	0.026
517	90	6	Severe	3	200	Imported	4.324	0.026
518	70	18	Severe	2	0	Imported	4.323	0.026
519	90	6	Severe	1	400	Imported	4.322	0.026
520	70	18	Severe	2	200	Imported	4.321	0.026
521	90	6	Severe	2	600	Imported	4.32	0.026
522	90	6	Severe	3	600	Imported	4.32	0.026
523	70	18	Severe	3	400	Imported	4.319	0.026
524	90	6	Severe	2	800	Imported	4.318	0.026
525	90	6	Severe	3	800	Imported	4.318	0.026
526	70	18	Severe	1	600	Imported	4.317	0.026
527	90	6	Severe	2	1000	Imported	4.316	0.026
528	70	18	Severe	1	800	Imported	4.315	0.026
529	70	18	Severe	2	800	Imported	4.315	0.026
530	50	6	No	2	0	Imported	4.23	0.024
531	50	6	No	1	600	Imported	4.224	0.024
532	50	18	Moderate	2	0	Domestic	4.2	0.023
533	50	18	Moderate	3	0	Domestic	4.2	0.023
534	50	18	Moderate	3	200	Domestic	4.198	0.023
535	50	18	Moderate	1	600	Domestic	4.194	0.023
536	50	18	Moderate	2	600	Domestic	4.194	0.023
537	50	18	Moderate	1	800	Domestic	4.192	0.023
538	50	18	Moderate	2	800	Domestic	4.192	0.023
539	50	18	Moderate	2	1000	Domestic	4.19	0.023
540	70	18	No	1	0	Domestic	4.187	0.023
541	70	18	No	2	0	Domestic	4.187	0.023
542	70	18	No	1	200	Domestic	4.185	0.023
543	70	18	No	3	200	Domestic	4.185	0.023
544	70	18	No	3	400	Domestic	4.183	0.023
545	70	18	No	3	600	Domestic	4.181	0.023
546	70	18	No	1	1000	Domestic	4.177	0.023
547	50	12	Moderate	1	400	Imported	4.146	0.022
548	50	12	Moderate	3	400	Imported	4.146	0.022
549	70	18	Severe	1	0	Domestic	4.145	0.022
550	70	18	Severe	3	0	Domestic	4.145	0.022
551	50	12	Moderate	1	600	Imported	4.144	0.022
552	90	6	Severe	2	400	Domestic	4.144	0.022
553	90	6	Severe	3	400	Domestic	4.144	0.022
554	90	6	Severe	1	600	Domestic	4.142	0.022
555	70	18	Severe	1	400	Domestic	4.141	0.022
556	70	18	Severe	2	400	Domestic	4.141	0.022
557	50	12	Moderate	2	1000	Imported	4.14	0.022
558	90	6	Severe	1	800	Domestic	4.14	0.022
559	70	18	Severe	3	600	Domestic	4.139	0.022
560	90	6	Severe	1	1000	Domestic	4.138	0.022
561	70	12	No	1	0	Imported	4.137	0.022
562	70	12	No	3	0	Imported	4.137	0.022
563	70	12	No	1	600	Imported	4.131	0.022
564	70	12	No	1	1000	Imported	4.127	0.022
565	70	12	Severe	2	0	Imported	4.095	0.021
566	70	12	Severe	3	0	Imported	4.095	0.021
567	70	12	Severe	3	200	Imported	4.093	0.021
568	70	12	Severe	1	400	Imported	4.091	0.021
569	70	12	Severe	1	1000	Imported	4.085	0.021
570	70	12	Severe	3	1000	Imported	4.085	0.021
571	70	6	Moderate	3	0	Imported	4.075	0.021
572	70	6	Moderate	3	200	Imported	4.073	0.02
573	70	6	Moderate	2	600	Imported	4.069	0.02
574	70	6	Moderate	1	800	Imported	4.067	0.02
575	70	6	Moderate	2	800	Imported	4.067	0.02
576	70	6	Moderate	3	800	Imported	4.067	0.02
577	70	6	Moderate	1	1000	Imported	4.065	0.02
578	50	6	No	1	0	Domestic	4.052	0.02
579	50	6	No	3	0	Domestic	4.052	0.02
580	50	6	No	1	400	Domestic	4.048	0.02
581	50	6	No	2	600	Domestic	4.046	0.02
582	50	6	No	3	600	Domestic	4.046	0.02
583	50	6	No	2	800	Domestic	4.044	0.02
584	50	6	No	3	800	Domestic	4.044	0.02
585	50	6	No	3	1000	Domestic	4.042	0.02
586	50	12	Moderate	1	0	Domestic	3.972	0.018
587	50	12	Moderate	1	200	Domestic	3.97	0.018
588	50	12	Moderate	2	200	Domestic	3.97	0.018
589	50	12	Moderate	2	400	Domestic	3.968	0.018
590	50	12	Moderate	2	800	Domestic	3.964	0.018
591	50	12	Moderate	1	1000	Domestic	3.962	0.018
592	50	12	Moderate	3	1000	Domestic	3.962	0.018
593	70	12	No	3	400	Domestic	3.955	0.018
594	70	12	No	3	600	Domestic	3.953	0.018
595	50	18	No	3	0	Imported	3.949	0.018
596	70	12	No	2	1000	Domestic	3.949	0.018
597	50	18	No	1	200	Imported	3.947	0.018
598	50	18	No	1	400	Imported	3.945	0.018
599	50	18	No	2	400	Imported	3.945	0.018
600	50	18	No	1	800	Imported	3.941	0.018
601	50	18	No	3	800	Imported	3.941	0.018
602	50	18	No	1	1000	Imported	3.939	0.018
603	50	18	No	3	1000	Imported	3.939	0.018
604	70	12	Severe	1	200	Domestic	3.915	0.017
605	70	12	Severe	2	400	Domestic	3.913	0.017
606	70	12	Severe	3	400	Domestic	3.913	0.017
607	70	12	Severe	2	600	Domestic	3.911	0.017
608	70	12	Severe	2	800	Domestic	3.909	0.017
609	70	12	Severe	3	800	Domestic	3.909	0.017
610	70	12	Severe	2	1000	Domestic	3.907	0.017
611	50	18	Severe	2	200	Imported	3.905	0.017
612	50	18	Severe	1	400	Imported	3.903	0.017
613	50	18	Severe	1	600	Imported	3.901	0.017
614	50	18	Severe	2	800	Imported	3.899	0.017
615	50	18	Severe	2	1000	Imported	3.897	0.017
616	70	6	Moderate	1	0	Domestic	3.897	0.017
617	70	6	Moderate	2	0	Domestic	3.897	0.017
618	70	6	Moderate	1	200	Domestic	3.895	0.017
619	70	6	Moderate	1	400	Domestic	3.893	0.017
620	70	6	Moderate	2	400	Domestic	3.893	0.017
621	70	6	Moderate	3	400	Domestic	3.893	0.017
622	70	6	Moderate	1	600	Domestic	3.891	0.017
623	70	18	Moderate	1	200	Imported	3.792	0.015
624	70	18	Moderate	2	200	Imported	3.792	0.015
625	70	18	Moderate	3	200	Imported	3.792	0.015
626	70	18	Moderate	1	400	Imported	3.79	0.015
627	70	18	Moderate	2	400	Imported	3.79	0.015
628	70	18	Moderate	1	600	Imported	3.788	0.015
629	70	18	Moderate	1	800	Imported	3.786	0.015
630	70	18	Moderate	1	1000	Imported	3.784	0.015
631	70	18	Moderate	2	1000	Imported	3.784	0.015
632	50	18	No	1	0	Domestic	3.771	0.015
633	50	18	No	2	0	Domestic	3.771	0.015
634	50	18	No	3	200	Domestic	3.769	0.015
635	50	18	No	2	800	Domestic	3.763	0.015
636	50	18	Severe	1	0	Domestic	3.729	0.015
637	50	18	Severe	1	200	Domestic	3.727	0.014
638	50	18	Severe	3	200	Domestic	3.727	0.014
639	50	18	Severe	2	400	Domestic	3.725	0.014
640	50	18	Severe	2	600	Domestic	3.723	0.014
641	50	18	Severe	3	600	Domestic	3.723	0.014
642	50	12	No	1	0	Imported	3.721	0.014
643	50	18	Severe	3	800	Domestic	3.721	0.014
644	50	18	Severe	1	1000	Domestic	3.719	0.014
645	50	12	No	1	600	Imported	3.715	0.014
646	50	12	No	3	600	Imported	3.715	0.014
647	50	12	No	1	800	Imported	3.713	0.014
648	50	12	No	2	800	Imported	3.713	0.014
649	50	12	No	3	800	Imported	3.713	0.014
650	50	12	No	2	1000	Imported	3.711	0.014
651	50	12	Severe	3	0	Imported	3.679	0.014
652	50	12	Severe	3	200	Imported	3.677	0.014
653	50	12	Severe	2	800	Imported	3.671	0.014
654	50	12	Severe	3	800	Imported	3.671	0.014
655	50	12	Severe	3	1000	Imported	3.669	0.014
656	50	6	Moderate	3	200	Imported	3.657	0.013
657	50	6	Moderate	1	400	Imported	3.655	0.013
658	50	6	Moderate	2	400	Imported	3.655	0.013
659	50	6	Moderate	2	600	Imported	3.653	0.013
660	50	6	Moderate	1	800	Imported	3.651	0.013
661	50	6	Moderate	3	800	Imported	3.651	0.013
662	50	6	Moderate	1	1000	Imported	3.649	0.013
663	50	6	Moderate	3	1000	Imported	3.649	0.013
664	70	6	No	1	0	Imported	3.646	0.013
665	70	6	No	2	200	Imported	3.644	0.013
666	70	6	No	3	200	Imported	3.644	0.013
667	70	6	No	2	400	Imported	3.642	0.013
668	70	6	No	3	400	Imported	3.642	0.013
669	70	6	No	2	600	Imported	3.64	0.013
670	70	6	No	2	800	Imported	3.638	0.013
671	70	6	No	1	1000	Imported	3.636	0.013
672	70	6	No	2	1000	Imported	3.636	0.013
673	70	6	No	3	1000	Imported	3.636	0.013
674	70	18	Moderate	1	0	Domestic	3.616	0.013
675	70	18	Moderate	2	0	Domestic	3.616	0.013
676	70	6	Severe	2	0	Imported	3.604	0.013
677	70	6	Severe	2	200	Imported	3.602	0.013
678	70	6	Severe	1	400	Imported	3.6	0.013
679	70	6	Severe	2	800	Imported	3.596	0.013
680	70	6	Severe	3	800	Imported	3.596	0.013
681	70	12	Moderate	2	200	Imported	3.564	0.012
682	70	12	Moderate	3	200	Imported	3.564	0.012
683	70	12	Moderate	2	400	Imported	3.562	0.012
684	70	12	Moderate	1	600	Imported	3.56	0.012
685	70	12	Moderate	3	600	Imported	3.56	0.012
686	70	12	Moderate	1	800	Imported	3.558	0.012
687	70	12	Moderate	3	800	Imported	3.558	0.012
688	70	12	Moderate	1	1000	Imported	3.556	0.012
689	70	12	Moderate	3	1000	Imported	3.556	0.012
690	50	12	No	2	0	Domestic	3.543	0.012
691	50	12	No	3	0	Domestic	3.543	0.012
692	50	12	No	2	200	Domestic	3.541	0.012
693	50	12	No	2	400	Domestic	3.539	0.012
694	50	12	No	3	400	Domestic	3.539	0.012
695	50	12	No	2	600	Domestic	3.537	0.012
696	50	12	Severe	1	0	Domestic	3.501	0.012
697	50	12	Severe	2	0	Domestic	3.501	0.012
698	50	12	Severe	2	200	Domestic	3.499	0.012
699	50	12	Severe	1	400	Domestic	3.497	0.012
700	50	12	Severe	2	400	Domestic	3.497	0.012
701	50	12	Severe	3	400	Domestic	3.497	0.012
702	50	12	Severe	2	600	Domestic	3.495	0.011
703	50	12	Severe	3	600	Domestic	3.495	0.011
704	50	12	Severe	1	1000	Domestic	3.491	0.011
705	50	6	Moderate	1	0	Domestic	3.481	0.011
706	50	6	Moderate	2	0	Domestic	3.481	0.011
707	50	6	Moderate	1	200	Domestic	3.479	0.011
708	50	6	Moderate	2	200	Domestic	3.479	0.011
709	50	6	Moderate	3	400	Domestic	3.477	0.011
710	50	6	Moderate	3	600	Domestic	3.475	0.011
711	50	6	Moderate	2	1000	Domestic	3.471	0.011
712	70	6	No	2	0	Domestic	3.468	0.011
713	70	6	No	3	0	Domestic	3.468	0.011
714	70	6	No	1	200	Domestic	3.466	0.011
715	70	6	No	1	400	Domestic	3.464	0.011
716	70	6	No	1	600	Domestic	3.462	0.011
717	70	6	Severe	1	0	Domestic	3.426	0.011
718	70	6	Severe	3	200	Domestic	3.424	0.011
719	70	6	Severe	2	400	Domestic	3.422	0.011
720	70	6	Severe	1	600	Domestic	3.42	0.011
721	70	6	Severe	2	600	Domestic	3.42	0.011
722	70	6	Severe	3	600	Domestic	3.42	0.011
723	70	12	Moderate	1	200	Domestic	3.386	0.01
724	70	12	Moderate	1	400	Domestic	3.384	0.01
725	70	12	Moderate	3	400	Domestic	3.384	0.01
726	70	12	Moderate	2	600	Domestic	3.382	0.01
727	70	12	Moderate	2	800	Domestic	3.38	0.01
728	50	18	Moderate	3	0	Imported	3.378	0.01
729	70	12	Moderate	2	1000	Domestic	3.378	0.01
730	50	18	Moderate	1	400	Imported	3.374	0.01
731	50	18	Moderate	2	400	Imported	3.374	0.01
732	50	18	Moderate	2	1000	Imported	3.368	0.01
733	70	18	Severe	3	0	Imported	3.323	0.01
734	70	18	Severe	3	200	Imported	3.321	0.01
735	70	18	Severe	1	400	Imported	3.319	0.01
736	70	18	Severe	3	600	Imported	3.317	0.01
737	70	18	Severe	3	800	Imported	3.315	0.01
738	70	18	Severe	2	1000	Imported	3.313	0.01
739	50	6	No	1	0	Imported	3.23	0.009
740	50	6	No	2	400	Imported	3.226	0.009
741	50	6	No	3	400	Imported	3.226	0.009
742	50	6	No	1	800	Imported	3.222	0.009
743	50	6	No	1	1000	Imported	3.22	0.009
744	50	6	No	3	1000	Imported	3.22	0.009
745	50	18	Moderate	1	0	Domestic	3.2	0.009
746	50	18	Moderate	1	200	Domestic	3.198	0.009
747	50	18	Moderate	2	200	Domestic	3.198	0.009
748	50	18	Moderate	3	400	Domestic	3.196	0.009
749	50	18	Moderate	3	600	Domestic	3.194	0.008
750	50	18	Moderate	3	800	Domestic	3.192	0.008
751	50	18	Moderate	1	1000	Domestic	3.19	0.008
752	50	18	Moderate	3	1000	Domestic	3.19	0.008
753	50	6	Severe	2	200	Imported	3.186	0.008
754	50	6	Severe	2	800	Imported	3.18	0.008
755	50	12	Moderate	1	0	Imported	3.15	0.008
756	50	12	Moderate	3	0	Imported	3.15	0.008
757	50	12	Moderate	1	200	Imported	3.148	0.008
758	50	12	Moderate	3	200	Imported	3.148	0.008
759	50	12	Moderate	2	400	Imported	3.146	0.008
760	70	18	Severe	1	200	Domestic	3.143	0.008
761	50	12	Moderate	1	800	Imported	3.142	0.008
762	50	12	Moderate	3	800	Imported	3.142	0.008
763	70	18	Severe	2	600	Domestic	3.139	0.008
764	70	18	Severe	1	800	Domestic	3.137	0.008
765	70	18	Severe	2	800	Domestic	3.137	0.008
766	70	18	Severe	1	1000	Domestic	3.135	0.008
767	70	18	Severe	3	1000	Domestic	3.135	0.008
768	70	12	Severe	1	0	Imported	3.095	0.008
769	70	12	Severe	1	200	Imported	3.093	0.008
770	70	12	Severe	2	200	Imported	3.093	0.008
771	70	12	Severe	1	600	Imported	3.089	0.008
772	70	12	Severe	2	600	Imported	3.089	0.008
773	70	12	Severe	1	800	Imported	3.087	0.008
774	70	12	Severe	3	800	Imported	3.087	0.008
775	70	6	Moderate	1	200	Imported	3.073	0.008
776	70	6	Moderate	2	400	Imported	3.071	0.008
777	70	6	Moderate	3	1000	Imported	3.065	0.007
778	50	6	No	2	0	Domestic	3.052	0.007
779	50	6	No	1	200	Domestic	3.05	0.007
780	50	6	No	2	200	Domestic	3.05	0.007
781	50	6	No	3	200	Domestic	3.05	0.007
782	50	6	No	2	1000	Domestic	3.042	0.007
783	50	6	Severe	2	0	Domestic	3.01	0.007
784	50	6	Severe	1	200	Domestic	3.008	0.007
785	50	6	Severe	3	200	Domestic	3.008	0.007
786	50	6	Severe	3	400	Domestic	3.006	0.007
787	50	6	Severe	1	600	Domestic	3.004	0.007
788	50	6	Severe	1	800	Domestic	3.002	0.007
789	50	6	Severe	3	800	Domestic	3.002	0.007
790	50	12	Moderate	2	0	Domestic	2.972	0.007
791	50	12	Moderate	1	400	Domestic	2.968	0.007
792	50	12	Moderate	3	400	Domestic	2.968	0.007
793	50	12	Moderate	2	600	Domestic	2.966	0.007
794	50	12	Moderate	3	600	Domestic	2.966	0.007
795	50	18	No	1	0	Imported	2.949	0.007
796	50	18	No	3	200	Imported	2.947	0.007
797	50	18	No	3	400	Imported	2.945	0.007
798	50	18	No	2	600	Imported	2.943	0.007
799	50	18	No	2	800	Imported	2.941	0.007
800	50	18	No	2	1000	Imported	2.939	0.007
801	70	12	Severe	1	400	Domestic	2.913	0.006
802	70	12	Severe	3	600	Domestic	2.911	0.006
803	50	18	Severe	1	0	Imported	2.907	0.006
804	50	18	Severe	2	0	Imported	2.907	0.006
805	50	18	Severe	3	0	Imported	2.907	0.006
806	50	18	Severe	1	200	Imported	2.905	0.006
807	50	18	Severe	2	400	Imported	2.903	0.006
808	50	18	Severe	2	600	Imported	2.901	0.006
809	50	18	Severe	3	800	Imported	2.899	0.006
810	50	18	Severe	1	1000	Imported	2.897	0.006
811	70	6	Moderate	2	200	Domestic	2.895	0.006
812	70	6	Moderate	3	600	Domestic	2.891	0.006
813	70	6	Moderate	2	800	Domestic	2.889	0.006
814	70	6	Moderate	3	800	Domestic	2.889	0.006
815	70	6	Moderate	2	1000	Domestic	2.887	0.006
816	50	18	No	2	200	Domestic	2.769	0.006
817	50	18	No	1	400	Domestic	2.767	0.006
818	50	18	No	2	400	Domestic	2.767	0.006
819	50	18	No	1	600	Domestic	2.765	0.006
820	50	18	No	3	600	Domestic	2.765	0.006
821	50	18	No	3	800	Domestic	2.763	0.006
822	50	18	No	3	1000	Domestic	2.761	0.006
823	50	18	Severe	1	400	Domestic	2.725	0.005
824	50	18	Severe	3	400	Domestic	2.725	0.005
825	50	18	Severe	1	800	Domestic	2.721	0.005
826	50	18	Severe	2	800	Domestic	2.721	0.005
827	50	12	No	1	200	Imported	2.719	0.005
828	50	12	No	2	200	Imported	2.719	0.005
829	50	18	Severe	3	1000	Domestic	2.719	0.005
830	50	12	No	1	400	Imported	2.717	0.005
831	50	12	No	3	400	Imported	2.717	0.005
832	50	12	No	1	1000	Imported	2.711	0.005
833	50	12	Severe	2	200	Imported	2.677	0.005
834	50	12	Severe	2	400	Imported	2.675	0.005
835	50	12	Severe	3	400	Imported	2.675	0.005
836	50	12	Severe	3	600	Imported	2.673	0.005
837	50	12	Severe	1	800	Imported	2.671	0.005
838	50	6	Moderate	1	0	Imported	2.659	0.005
839	50	6	Moderate	2	0	Imported	2.659	0.005
840	50	6	Moderate	3	0	Imported	2.659	0.005
841	50	6	Moderate	2	200	Imported	2.657	0.005
842	50	6	Moderate	3	400	Imported	2.655	0.005
843	50	6	Moderate	3	600	Imported	2.653	0.005
844	50	6	Moderate	2	1000	Imported	2.649	0.005
845	70	6	Severe	1	0	Imported	2.604	0.005
846	70	6	Severe	3	0	Imported	2.604	0.005
847	70	6	Severe	1	200	Imported	2.602	0.005
848	70	6	Severe	3	200	Imported	2.602	0.005
849	70	6	Severe	3	400	Imported	2.6	0.005
850	70	6	Severe	1	600	Imported	2.598	0.005
851	70	6	Severe	2	600	Imported	2.598	0.005
852	70	6	Severe	3	600	Imported	2.598	0.005
853	70	6	Severe	1	800	Imported	2.596	0.005
854	70	6	Severe	2	1000	Imported	2.594	0.005
855	70	6	Severe	3	1000	Imported	2.594	0.005
856	50	12	No	1	0	Domestic	2.543	0.004
857	50	12	No	3	200	Domestic	2.541	0.004
858	50	12	No	3	800	Domestic	2.535	0.004
859	50	12	No	3	1000	Domestic	2.533	0.004
860	50	12	Severe	3	0	Domestic	2.501	0.004
861	50	12	Severe	1	200	Domestic	2.499	0.004
862	50	12	Severe	3	200	Domestic	2.499	0.004
863	50	12	Severe	1	600	Domestic	2.495	0.004
864	50	12	Severe	2	800	Domestic	2.493	0.004
865	50	12	Severe	3	800	Domestic	2.493	0.004
866	50	12	Severe	2	1000	Domestic	2.491	0.004
867	50	6	Moderate	1	400	Domestic	2.477	0.004
868	50	6	Moderate	2	400	Domestic	2.477	0.004
869	50	6	Moderate	1	600	Domestic	2.475	0.004
870	50	6	Moderate	1	800	Domestic	2.473	0.004
871	50	6	Moderate	2	800	Domestic	2.473	0.004
872	50	6	Moderate	3	800	Domestic	2.473	0.004
873	50	6	Moderate	1	1000	Domestic	2.471	0.004
874	70	6	Severe	2	0	Domestic	2.426	0.004
875	70	6	Severe	2	200	Domestic	2.424	0.004
876	70	6	Severe	2	800	Domestic	2.418	0.004
877	70	6	Severe	3	800	Domestic	2.418	0.004
878	70	6	Severe	1	1000	Domestic	2.416	0.004
879	50	18	Moderate	2	0	Imported	2.378	0.004
880	50	18	Moderate	1	200	Imported	2.376	0.004
881	50	18	Moderate	2	200	Imported	2.376	0.004
882	50	18	Moderate	3	200	Imported	2.376	0.004
883	50	18	Moderate	1	600	Imported	2.372	0.004
884	50	18	Moderate	2	600	Imported	2.372	0.004
885	50	18	Moderate	3	600	Imported	2.372	0.004
886	50	18	Moderate	1	800	Imported	2.37	0.004
887	50	18	Moderate	2	800	Imported	2.37	0.004
888	50	18	Moderate	1	1000	Imported	2.368	0.004
889	50	6	No	3	0	Imported	2.23	0.003
890	50	6	No	1	200	Imported	2.228	0.003
891	50	6	No	2	200	Imported	2.228	0.003
892	50	6	No	3	200	Imported	2.228	0.003
893	50	6	No	1	400	Imported	2.226	0.003
894	50	6	No	2	600	Imported	2.224	0.003
895	50	6	No	3	600	Imported	2.224	0.003
896	50	6	No	2	800	Imported	2.222	0.003
897	50	6	No	3	800	Imported	2.222	0.003
898	50	6	No	2	1000	Imported	2.22	0.003
899	50	18	Moderate	1	400	Domestic	2.196	0.003
900	50	18	Moderate	2	400	Domestic	2.196	0.003
901	50	6	Severe	1	0	Imported	2.188	0.003
902	50	6	Severe	3	0	Imported	2.188	0.003
903	50	6	Severe	2	400	Imported	2.184	0.003
904	50	6	Severe	2	600	Imported	2.182	0.003
905	50	6	Severe	1	800	Imported	2.18	0.003
906	50	12	Moderate	2	0	Imported	2.15	0.003
907	50	12	Moderate	2	200	Imported	2.148	0.003
908	50	12	Moderate	2	600	Imported	2.144	0.003
909	50	12	Moderate	3	600	Imported	2.144	0.003
910	50	12	Moderate	2	800	Imported	2.142	0.003
911	50	12	Moderate	1	1000	Imported	2.14	0.003
912	50	12	Moderate	3	1000	Imported	2.14	0.003
913	50	6	No	2	400	Domestic	2.048	0.003
914	50	6	No	3	400	Domestic	2.048	0.003
915	50	6	No	1	600	Domestic	2.046	0.003
916	50	6	No	1	800	Domestic	2.044	0.003
917	50	6	No	1	1000	Domestic	2.042	0.003
918	50	6	Severe	1	400	Domestic	2.006	0.003
919	50	6	Severe	3	600	Domestic	2.004	0.003
920	50	6	Severe	2	800	Domestic	2.002	0.003
921	50	6	Severe	1	1000	Domestic	2	0.003
922	50	6	Severe	2	1000	Domestic	2	0.003
923	50	6	Severe	3	1000	Domestic	2	0.003
924	50	12	Moderate	3	0	Domestic	1.972	0.003
925	50	12	Moderate	3	200	Domestic	1.97	0.002
926	50	12	Moderate	1	600	Domestic	1.966	0.002
927	50	12	Moderate	1	800	Domestic	1.964	0.002
928	50	12	Moderate	3	800	Domestic	1.964	0.002
929	50	12	Moderate	2	1000	Domestic	1.962	0.002
930	50	18	Severe	3	200	Imported	1.905	0.002
931	50	18	Severe	3	400	Imported	1.903	0.002
932	50	18	Severe	3	600	Imported	1.901	0.002
933	50	18	Severe	1	800	Imported	1.899	0.002
934	50	18	Severe	3	1000	Imported	1.897	0.002
935	50	18	Severe	2	0	Domestic	1.729	0.002
936	50	18	Severe	3	0	Domestic	1.729	0.002
937	50	18	Severe	2	200	Domestic	1.727	0.002
938	50	18	Severe	1	600	Domestic	1.723	0.002
939	50	18	Severe	2	1000	Domestic	1.719	0.002
940	50	12	Severe	1	0	Imported	1.679	0.002
941	50	12	Severe	2	0	Imported	1.679	0.002
942	50	12	Severe	1	200	Imported	1.677	0.002
943	50	12	Severe	1	400	Imported	1.675	0.002
944	50	12	Severe	1	600	Imported	1.673	0.002
945	50	12	Severe	2	600	Imported	1.673	0.002
946	50	12	Severe	1	1000	Imported	1.669	0.002
947	50	12	Severe	2	1000	Imported	1.669	0.002
948	50	6	Moderate	1	200	Imported	1.657	0.002
949	50	6	Moderate	1	600	Imported	1.653	0.002
950	50	6	Moderate	2	800	Imported	1.651	0.002
951	50	12	Severe	1	800	Domestic	1.493	0.002
952	50	12	Severe	3	1000	Domestic	1.491	0.002
953	50	6	Moderate	3	0	Domestic	1.481	0.002
954	50	6	Moderate	3	200	Domestic	1.479	0.002
955	50	6	Moderate	2	600	Domestic	1.475	0.002
956	50	6	Moderate	3	1000	Domestic	1.471	0.002
957	50	6	Severe	2	0	Imported	1.188	0.001
958	50	6	Severe	1	200	Imported	1.186	0.001
959	50	6	Severe	3	200	Imported	1.186	0.001
960	50	6	Severe	1	400	Imported	1.184	0.001
961	50	6	Severe	3	400	Imported	1.184	0.001
962	50	6	Severe	1	600	Imported	1.182	0.001
963	50	6	Severe	3	600	Imported	1.182	0.001
964	50	6	Severe	3	800	Imported	1.18	0.001
965	50	6	Severe	1	1000	Imported	1.178	0.001
966	50	6	Severe	2	1000	Imported	1.178	0.001
967	50	6	Severe	3	1000	Imported	1.178	0.001
968	50	6	Severe	1	0	Domestic	1.01	0.001
969	50	6	Severe	3	0	Domestic	1.01	0.001
970	50	6	Severe	2	200	Domestic	1.008	0.001
971	50	6	Severe	2	400	Domestic	1.006	0.001
972	50	6	Severe	2	600	Domestic	1.004	0.001

## Data Availability

The data that support the findings of this study are available from the corresponding authors upon reasonable request.
